# Structural insights into the C-terminus of the histone-lysine N-methyltransferase NSD3 by small-angle X-ray scattering

**DOI:** 10.3389/fmolb.2024.1191246

**Published:** 2024-03-07

**Authors:** Benny Danilo Belviso, Yunpeng Shen, Benedetta Carrozzini, Masayo Morishita, Eric di Luccio, Rocco Caliandro

**Affiliations:** ^1^ Institute of Crystallography, CNR, Bari, Italy; ^2^ Department of Biotechnology, School of Biological Engineering, Henan University of Technology, Zhengzhou, Henan, China; ^3^ Department of Genetic Engineering, School of Life Sciences, College of Natural Sciences, Kyungpook National University, Daegu, Republic of Korea

**Keywords:** nuclear receptor-binding SET domain protein 3, small-angle X-ray scattering, computational modeling, epigenetic cancer therapy, molecular dynamics

## Abstract

NSD3 is a member of six H3K36-specific histone lysine methyltransferases in metazoans. Its overexpression or mutation is implicated in developmental defects and oncogenesis. Aside from the well-characterized catalytic SET domain, NSD3 has multiple clinically relevant potential chromatin-binding motifs, such as the proline–tryptophan–tryptophan–proline (PWWP), the plant homeodomain (PHD), and the adjacent Cys-His-rich domain located at the C-terminus. The crystal structure of the individual domains is available, and this structural knowledge has allowed the designing of potential inhibitors, but the intrinsic flexibility of larger constructs has hindered the characterization of mutual domain conformations. Here, we report the first structural characterization of the NSD3 C-terminal region comprising the PWWP2, SET, and PHD4 domains, which has been achieved at a low resolution in solution by small-angle X-ray scattering (SAXS) data on two multiple-domain NSD3 constructs complemented with size-exclusion chromatography and advanced computational modeling. Structural models predicted by machine learning have been validated in direct space, by comparison with the SAXS-derived molecular envelope, and in reciprocal space, by reproducing the experimental SAXS profile. Selected models have been refined by SAXS-restrained molecular dynamics. This study shows how SAXS data can be used with advanced computational modeling techniques to achieve a detailed structural characterization and sheds light on how NSD3 domains are interconnected in the C-terminus.

## 1 Introduction

Nuclear receptor-binding SET domain (NSD) proteins are three protein lysine methyltransferases that are predominantly mono- and di-methylate lysine 36 of histone 3 (H3K36) (Kuo et al., 2011). They are called NSD1, NSD2 (also known as WHSC1 or MMSET), and NSD3 (also known as WHSC1L1) and are critical in maintaining chromatin integrity. Their overexpression or mutation is implicated in developmental defects and oncogenesis. In addition, the dysfunction of their methylation activity results in epigenomic aberrations, which are relevant for oncogenesis. Thus, reducing NSD activity through specific lysine-HMTase inhibitors appears promising for epigenetic cancer therapy (Vougiouklakis et al., 2015).

NSD2 is an oncoprotein that is aberrantly expressed, amplified, or somatically mutated in multiple types of cancer (Vougiouklakis et al., 2015). Notably, the t (4; 14) NSD2 translocation in multiple myeloma and the hyperactive NSD2 mutation E1099K in a subset of pediatric acute lymphoblastic leukemia result in altered chromatin methylation that drives oncogenesis (Keats et al., 2003; Jaffe et al., 2013). NSD3 is involved in several varieties of cancers as it contributes to tumorigenesis by interacting with the bromodomain-containing protein 4 (BRD4) and the bromodomain and extra-terminal (BET) protein, which are potential therapeutic targets in acute myeloid leukemia (Han et al., 2018).

NSD2 and NSD3 have multiple protein–protein interaction domains that may be clinically relevant and arranged in a conserved sequence that contains two proline–tryptophan–tryptophan–proline (PWWP) domains, which are assumed to be critical for binding to methylated H3-histone and the DNA molecule, four plant homeodomains (PHDs)—which appear essential for interactions with other methylated histones—an associated with SET (AWS) domain, a catalytic SET domain, and a post-SET domain—including a Cys-His-rich region (C5HCH) (Angrand et al., 2001).

The first PWWP domain (PWWP1) of NSD2 binds *in vitro* H3K36me2, presumably through a conserved aromatic cage composed of three orthogonally positioned aromatic side chains (Y233, W236, and F266) that can engage in cation−π and hydrophobic interactions with the ammonium group of the methylated lysine (Qin and Min, 2014). However, the contribution of the PWWP domains and the role in histone methylation of the aromatic residues in the cage mentioned above is not established yet. For example, the F266A mutation at the aromatic cage, known to inhibit cancer proliferation, appears to affect chromatin/NSD2 binding without significantly affecting H3K36 dimethylation (Sankaran et al., 2016). Studies have revealed that AWS, SET, and post-SET domains also play a critical role in recognizing and methylating molecular targets of histones H3 and H4 *in vitro*, particularly in the case of NSD3 (Morishita et al., 2014).

High-resolution structural knowledge of individual domains from X-ray crystallography is available for NSD2 and NSD3 and has been used to design small-molecule inhibitors. The crystal structure of the SET domain supported the design and characterization of N-alkyl sinefungin derivatives for NSD2 (Tisi et al., 2016) and a norleucine-containing inhibitor peptide derived from the histone H4 sequence for NSD3 (Morrison et al., 2018). The crystal structure of the NSD2-PWWP1 enabled both the discovery of a small-molecule antagonist with a Kd of 3.4 μM, which abrogates histones containing H3K36me2 binding in cells (de Freitas et al., 2021), and the characterization of its interactions with methylated histone peptides and dsDNA (Zhang et al., 2021). Moreover, the crystal structure of PWWP1 of NSD3 allowed a fragment-based discovery of a potent, selective, and cellular active antagonist (Bottcher et al., 2019). Binding assay studies of the region, including the PHD closest to the C-terminus and the C5HCH motif of the NSD3, along with the crystal structure of such regions, revealed a histone-binding specificity of the PHD domain between the three members of the NSD family (He et al., 2013). Recently, cryo-electron microscopy has made available structures of the SET domain for NSD2 and NSD3 bound to mononucleosomes (Li et al., 2021; Sato et al., 2021), thus providing molecular insights into nucleosome-based recognition and histone-modification mechanisms.

Although both NSD2 and NSD3 are attractive therapeutic targets, efforts to target their domains with small-molecule inhibitors have so far met with little success (Morishita et al., 2017; Shen et al., 2019). On the other hand, drug design initiatives targeting NSD2 and NSD3 have been severely hampered by the lack of structural knowledge about mutual interactions between domains. The high-resolution structure of NSD2 or NSD3 constructs comprising PWWP, SET, and PHD domains is still missing, likely due to the high flexibility of these proteins that make them recalcitrant to obtain good-quality crystals for the structural solution by X-ray diffraction.

Here, we present the first structural investigation of the C-terminal region of NSD3, comprising the second PWWP domain (PWWP2), the SET domain, and the PHD closest to the C-terminus (PHD4), in solution, determined by small-angle X-ray scattering (SAXS) combined with advanced computational modeling. In particular, the molecular envelope determinations from SAXS data were complemented with structural predictions based on artificial intelligence, which is in line with a recent trend in the field of SAXS data analysis (Receveur-Bréchot, 2023), and with a molecular dynamics flexible-fitting approach, which has recently proven effective even for highly flexible proteins (Belviso et al., 2022). The mutual conformation of interacting domains in solution, thus not affected by the typical artifacts due to sample preparation for X-ray diffraction and cryo-EM, i.e., crystal packing or vitrification effects, respectively, was disclosed.

## 2 Materials and methods

### 2.1 NSD3 construct expression and purification

Two constructs for the C-terminal region of the NSD3 (UniProt code Q9BZ95) protein were designed: the first including PWWP2, AWS, SET, and PostSET domains, comprising residues from 942 to 1,318, and named NSD3-PWWP2-SET, and the second including AWS, SET, PostSET, and PHD4 domains, comprising residues from 1,070 to 1,423, and named NSD3-SET-PHD4. The conformed pTYB12-NSD construct plasmids were transformed into *Escherichia coli* BL21 (DE3) cells. The culture was incubated in an LB medium containing 100 mg/L ampicillin at 37°C, 180 rpm, until OD_600_ reached around 0.6. Then, 125 μM isopropyl 1-thio-D-galactopyranoside (IPTG) was added to induce the recombinant expression of the target construct proteins for 16 h at 12°C. Cells were harvested and frozen at −80°C for 2 h minimum. The frozen cells were re-suspended and lysed (shaking) for 30 min in IMPACT buffer (500 mM NaCl, 20 mM Tris pH 8.0, and 0.1 mM EDTA) with 0.1% Triton and 10 mM phenylmethanesulfonyl fluoride (PMSF), followed by 20 cycles of sonication (2.5 min at 85 Amp) on ice. After removing the cell debris, the lysate containing CBD (chitin-binding domain)-intein-target protein was loaded onto a chitin resin column and then flashed with 1 L IMPACT buffer with 0.1% Triton X-100 (45–60 column volumes) to remove other proteins and impurities and 0.5 L IMPACT buffer (25–30 column volumes) to remove the detergent Triton. Cleavage of the intein tag was induced by incubation in IMPACT buffer supplemented with 50 mM 2-mercaptoethanol at 4°C for 40 h. The pure target protein was eluted in 65 mL IMPACT buffer, concentrated, and washed with IMPACT buffer using 10K Amicon Ultra centrifugal filters.

### 2.2 SAXS measurements

Small-angle X-ray scattering (SAXS) measurements were performed at the beamline B21 of the Diamond Light Source (Didcot, UK), a beamline devoted to bioSAXS measurements and equipped with an EIGER 4M detector (Dectris) and in-line size-exclusion chromatography (SEC-SAXS). Protein samples were buffer exchanged against 0.5 M NaCl, 20 mM Tris-HCl (pH 8.5), and 5 mM DTT using an Amicon-4 Centrifugation Unit (cutoff 10 kDa) and concentrated up to 4.3 mg/mL just before data collection to avoid sample aggregation and/or degradation. The protein concentration was determined using a NanoDrop spectrophotometer Thermo 2000c. For both constructs, the extinction coefficient (ε) and molecular weight (MW) were calculated by the ExPASy ProtParam server (Gasteiger et al., 2005) based on their sequence (Supplementary Table S1). SEC-SAXS data collections were performed at 20°C by loading 50 μL of the sample onto a 4.6-mL high-performance Shodex 403 chromatographic column (10–700 kDa MW resolution range) connected to an Agilent 1200 HPLC system (Waters) and equilibrated with the same buffer as that used for the buffer-exchange step. Three different sample concentrations were loaded on the column (0.6, 1.6, and 3.8 mg/mL in the case of NSD3-PWWP2-SET and 1.0, 1.6, and 4.3 mg/mL in the case of NSD3-SET-PHD4), each prepared by diluting the protein stock solutions concentrated at 4.3 mg/mL. For such measurements, the integration time per frame was set to 3 s, and data were collected in the range of momentum transfer (*q*) from 0.0026 to 0.340 Å^-1^.

### 2.3 SAXS data analysis

Raw SAXS 2D images were processed by the DAWN processing pipeline (Wilhelm et al. 2027) to produce normalized and radially integrated SAXS curves. They were processed by SCÅTTER (Rambo, 2017) to yield chromatograms and *R*_*g*_ value estimates. Background subtraction and Guinier analysis were performed by the program PRIMUS of the ATSAS package (Manalastas-Cantos et al., 2021). The FIND_Dmax tool of SCÅTTER was used with the default parameters (suggested *D*_max_ and alpha ranges, Moore model, and usage of background information for *P(r)* determination) to estimate the best value of the maximum momentum transfer *q*-value (*q*_max_) to be used in data analysis (Tully et al., 2021). Original SAXS profiles were re-binned using the DATREGRID command of ATSAS to improve their signal-to-noise ratio and then to increase the *q*_max_ values.

The particle distance distribution function *P(r)* was determined using GNOM (Svergun, 1992) in the *q*-value range from the beginning of the Guinier region to *q*_max_ (Supplementary Table S2). The AMBIMETER program (Petroukhov and Svergun, 2015) was used to determine the number of shape topologies compatible with the *P(r)* curves and predict the uniqueness of the *ab initio* reconstructions.

*Ab initio* molecular envelope determination was performed on the best dataset for each construct, selected according to the values of *q*_max_ and the quality of the *P(r)* profile. A total of 20 models of the molecular envelope were generated for each dataset using the annealing procedure in the fast mode of the DAMMIF program (Franke and Svergun, 2009). They were spatially aligned based on the normalized spatial discrepancy calculated by the SUPCOMB program (Kozin and Svergun, 2001) and subsequently averaged, bead occupancy-weighted, and volume-corrected using DAMAVER (Volkov and Svergun, 2003). Additional refinement to the SAXS data using DAMMIN/DAMSTART in the slow mode (Svergun, 1999) was performed to generate a final dummy-atom representation of the shape and volume of each protein. The protein molecular mass was estimated from SAXS data using the consensus Bayesian assessment (Hajizadeh et al., 2018) implemented in the program PRIMUS.

### 2.4 Homology modeling

Homology modeling was performed following two strategies using SAXS data as the lever arm to adjust the structural predictions. In the first strategy, which follows a bottom–up approach, individual domains were independently generated and assembled *a posteriori* based on the agreement with SAXS data. Homology models of the following domains/regions belonging to the C-terminal region of NSD3 were generated by the Phyre2 server (Kelley et al., 2015): the core of the PWWP2 domain (942–1,025); the link connecting domains PWWP2 and SET (1,026–1,056); the region containing AWS, SET, and postSET (1,070–1,318); the core of the SET domain (1,070–1,289); the link connecting the SET and PHD4 domains (1,290–1,310); and the PHD4 domain (1,319–1,423). These models were manually placed into molecular envelopes calculated from the SEC-SAXS datasets to obtain starting models for rigid body fitting that has been performed by SASREF (Petoukhov and Svergun, 2005). In the second strategy, a structural prediction of the whole C-terminal region from PWWP2 to PHD4 was performed, following a top–down approach that ensures compatible modeling of the NSD3-PWWP-SET and the NSD3-SET-PHD4 constructs. In the first instance, the AlphaFold prediction about the whole NSD3 protein was downloaded from the AlphaFold protein structure database (Jumper et al., 2021), entry n. Q9BZ95, the fourth version of the model, was considered. In the second instance, ColabFold (Mirdita et al., 2022), RaptorX (Xu et al., 2021), and I-Tasser (Zheng et al., 2021) servers were used as the predictors, each supplying the five most probable structural models. They all make use of a machine learning approach; specifically, the first combines the fast homology search of MMseqs2 (Steinegger and Söding, 2017) with AlphaFold2 (Jumper et al., 2021) or RoseTTAFold (Baek et al., 2021), the second integrates deep learning and co-evolutionary analysis by means of convolutional residual neural networks, and the third combines contact maps from deep neural network learning with fragment assembly simulations. A mixed-strategy approach was also followed, where individual domains extracted from the AlphaFold prediction were used for SAXS-based rigid body modeling performed by the program CORAL (Petoukhov et al., 2012).

The quality of structural predictions has been assessed by comparison with SAXS data: each predicted model has been split in an NSD3-PWWP2-SET and NSD3-SET-PHD4 part, which has been separately fitted with SAXS data both in reciprocal and direct space. The validation parameter of the model in reciprocal space is the χ^2^ of the least-square fit with raw SAXS data, as determined by the CRYSOL program (Svergun et al., 1995), and that in direct space is the normalized spatial discrepancy with respect to the molecular envelope determined *ab initio* from SAXS data. This latter quantifier tends to be 0 for similar objects, is less than 1 among different DAMMIF/N model reconstructions of the same SAXS dataset, and is expected to be less than 3 when comparing SAXS-derived dummy-atom models with full-atom atomic models.

### 2.5 SAXS-driven optimization of structural models

The best-quality homology modeling models were subjected to molecular dynamics (MD) restrained by the SAXS-derived molecular envelope using the molecular dynamics flexible fitting (MDFF) tool (Trabuco et al., 2008), which implements the fitting of flexible atomic structures into a density map. The molecular envelopes determined by SEC-SAXS data were used as reference density maps, from which external potentials were generated and added to molecular dynamics. Simulations were performed by NAMD (NAnoscale Molecular Dynamics) (Phillips et al., 2020), and simulated data were analyzed by VMD (visual molecular dynamics) (Humphrey et al., 1996). MD simulations were run with an explicit solvent. Long-range electrostatic interactions were treated with the particle-mesh Ewald method (Darden et al., 1993). A 1.0 nm cutoff was used for van der Waals interactions and the real-space part of the electrostatic interactions. All bond lengths were constrained with the LINCS algorithm, and the time step was set to 1 fs. MDFF simulations were run with an implicit solvent, while targeted molecular dynamics (TMD) was used to maintain the internal consistency of the PWWP, SET, and PHD4 domains with respect to their experimental structures. Both the values of the dielectric constant and the scaling factor of the MD external potential generated from the SAXS density map were fine-tuned by optimizing the *a posteriori* agreement of the MD models with SAXS data. They were finally set to 100 and 0.08, respectively.

MDFF simulations were monitored by calculating the cross-correlation coefficient (CORR) between the target density map and each frame of the MDFF trajectory and the root-mean-square deviation of the C_α_ atoms (RMSD) for the initial structural model. The structural models were prepared for MD by setting the histidine protonation state to that expected at the pH used in the SAXS data collection (8.5), as predicted by the H++ server (Anandakrishnan et al., 2012), by adding Zn ions guided by their positions in the experimental models (four of them were positioned in the zinc-finger domain PHD4 and three in the SET domain) and deprotonating the closest cysteine residues to form expected S–S bonds. The metal coordination in the seven Zn sites was restrained using the NAMD extraBonds command, with a spring constant of 50 kcal/mol and a reference distance of 2.5 Å from Cys S or His N atoms.

MD trajectories were analyzed by extracting the region’s NSD3-PWWP2-SET and NSD3-SET-PHD4 from each frame and separately fitting them against SAXS data.

The structural models were compared using a descriptor based on the backbone dihedral angles. It is named the protein angular value (PAV) (Liuzzi et al., 2017), which is defined as follows:

$$PAV_{i}=\frac{180}{\pi }\cos^{-1}\left(\cos\left(\psi_{i}+\varphi_{i}\right)\right),$$ (1)

where *ψ*_*i*_ and *ϕ*_*i*_ are the backbone dihedral angles of the *i*th residue. The PAV values range between 0° and 180° and represent the *ψ*_*i*_+*ϕ*_*i*_ values expressed in degrees. Equation 1 avoids the problem of range definition connected with the circular nature of the angular variables. PAV profiles of each structure were calculated through the script TPAD (Caliandro et al., 2012) run on VMD (Humphrey et al., 1996). PAV profiles from different structures were separately analyzed using principal component analysis (PCA) and hierarchic clustering implemented in the program RootProf (Caliandro and Belviso, 2014).

Details about SAXS samples, data collection, analysis, and 3D modeling are summarized in Supplementary Table S2.

## 3 Results

### 3.1 Analysis of the SEC-SAXS data

**FIGURE 1:**
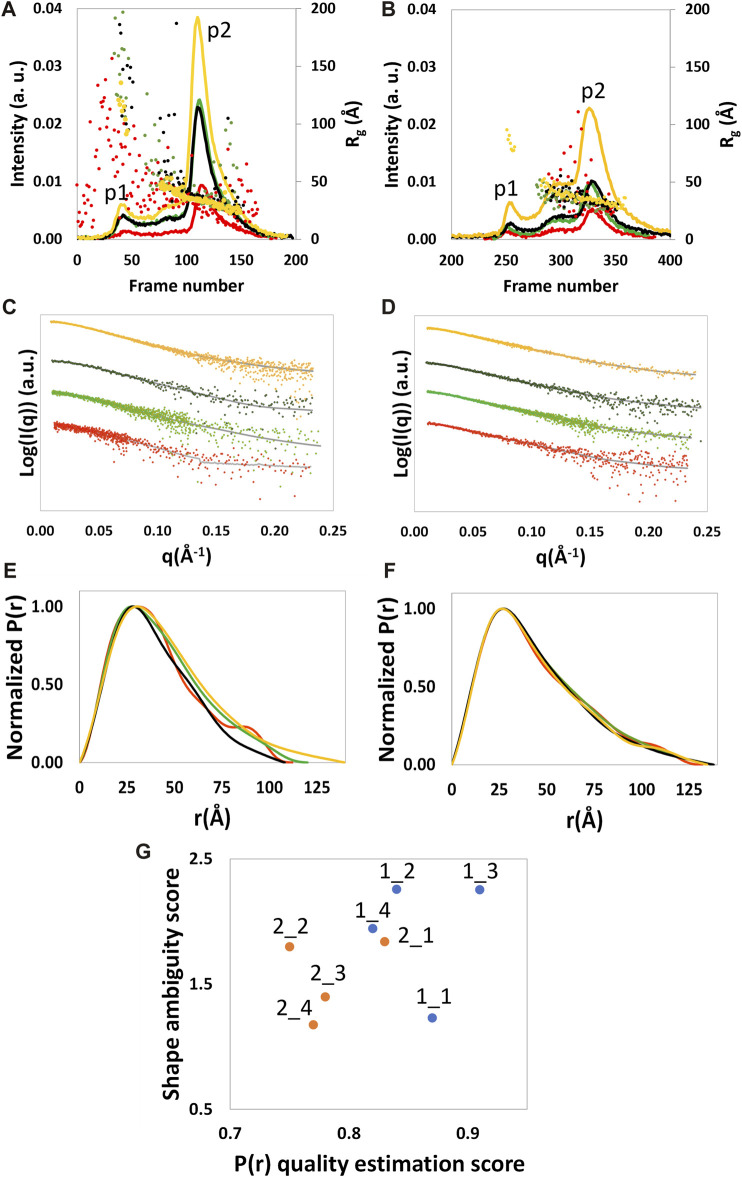
SEC profile and the *R*_*g*_ calculated by SCÅTTER for each frame **(A, B)**, experimental (dots) and calculated from the reciprocal space fit of *P(r)* to the data (full gray line) scattering intensity, with a scaled off set applied for presentation purposes **(C, D)**, and *P(r)* functions **(E, F)** are shown for NSD3-PWWP2-SET (first column) and NSD3-SET-PHD4 (second column) constructs. Red and yellow colors are used, respectively, for the samples at the lowest and higher protein concentrations, and green and black colors are used for the samples at 1.6 mg/mL. Correlation plot **(G)** between the shape ambiguity score, related to the number of shape topologies compatible with a given *P(r)* curve (vertical axis), and the quality score of the *P(r)* fit (horizontal axis). Points related to NSD3-PWWP2-SET and NSD3-SET-PHD4 are represented in orange and cyan color, respectively. Optimal values correspond to lower shape ambiguity (values lower than 1 correspond to potentially unique 3D reconstructions) and a higher quality score of the *P(r)* fit (maximum value is 1). Datasets 1_3, 1_4, 2_1, and 2_3 were linearly rebinned, and the others were log-rebinned.

SEC-SAXS analyzed NSD3-PWWP2-SET and NSD3-SET-PHD4 constructs at a concentration of protein loaded in the column of 0.6, 1.6, and 3.8 mg/mL for NSD3-PWWP2-SET and 1.0, 1.6, and 4.3 mg/mL for NSD3-SET-PHD4. A whitish precipitate appeared at higher protein concentrations, suggesting the onset of protein aggregation effects. SEC profiles and radius of gyration per frame (*R*_*g*_) are shown in Figures 1A and B. The presence of two peaks characterizes both SEC profiles, hereinafter named p1 (the peak at lower elution time) and p2 (the peak at higher elution time). SEC also shows a shoulder of p1 (at a lower elution time than the peak) for each dataset, which is particularly evident in the case of NSD3-PWWP2-SET (Figure 1A). However, a visual inspection of the *R*_*g*_ values suggests that only the p2 peak of both constructs is related to a homogeneous species and, therefore, is the only region of the chromatogram that is suitable for data analysis.

Frames under the p2 peak were selected for averaging using the standard deviation of the *R*_*g*_ values (*σ*_*<Rg>*_ in Supplementary Table S3). For each construct and protein concentration, we chose a set of adjacent frames that minimizes *σ*_*<Rg>*_ while keeping the number of frames as high as possible. The similarity among datasets of the same construct has been assessed by a reduced *χ* ^2^ statistic test, which showed that all datasets of the same construct are compatible with the same distribution (each pair of datasets shows a calculated *p*-value higher than a significance level α = 0.01 in Supplementary Figure S1). The lowest *p*-values (still higher than 0.01) were found while comparing the datasets at the lowest and highest protein concentrations, suggesting a lower probability that these datasets are comparable with each other concerning the other cases.

The Guinier analysis provided *R*_*g*_ values (in the reciprocal space) ranging from 31 to 34 Å for NSD3-PWWP2-SET and from 33 to 35 Å for NSD3-SET-PHD4 (Table 1). Regarding the maximum momentum transfer at which SAXS data analysis can be performed (*q*_max_), it is expected that its values increase with the protein concentration as a consequence of a higher signal-to-noise ratio. However, we found a non-negligible correlation only in the case of the NSD3-SET-PHD4 construct (Pearson coefficient = 0.6) (Table 1).

**TABLE 1:** Data and model parameters estimated for each dataset collected in the SEC-SAXS mode for NSD3-PWWP2-SET and NSD3-SET-PHD4 constructs. Protein concentration, maximum momentum transfer (*q*_max_) estimated before and after re-binning the data, radius of gyration (*R*_*g*_) from Guinier analysis (reciprocal space), *P(r)* function determination (real space), maximum inter-particle distance (*D*_max_), and molecular weight (*MW*) are shown.

				After re-binning						
Construct	ID	Protein concentration (mg/mL)	*q*_max_ (Å^-1^)	*q*_max_ (Å^-1^)	*R*_*g*_ (Å) reciprocal space	*R*_*g*_ (Å) direct space		*D*_max_ (Å)		*MW* (kDa)
NSD3-PWWP2-SET	1_4	3.8	0.16	0.23	36.7	36.8		139.4		53.1
	1_3	1.6	0.21	0.23	31.3	31.4		108.0		50.8
	1_2	1.6	0.13	0.24	34.1	34.2		120.0		48.7
	1_1	0.6	0.23	0.23	33.2	33.3		112.0		43.7
NSD3-SET-PHD4	2_4	4.3	0.20	0.29	36.2	36.3		134.6		42.8
	2_3	1.6	0.16	0.29	35.8	36.0		138.0		40.2
	2_2	1.6	0.14	0.30	36.7	36.8		137.0		40.2
	2_1	1.0	0.18	0.29	36.5	36.6		132.0		41.9

Given the limited resolution of available data (Table 1), we re-binned the SAXS profiles by reducing the number of points on a linear or a log scale in *q*. The degree of data reduction was optimized for each dataset based on the new value of *q*_max_ and the quality of the pair distance distribution function *P(r)* obtained. An example of the dependence of *q*_max_ on the number of points is given in Supplementary Figure S2. The re-binned profiles are shown in Figures 1C and D, and the corresponding *P(r)* curves were calculated for each dataset by selecting a range from the beginning of the Guinier region to *q*_max_ (Figures 1E and F). The related geometrical parameters (*R*_*g*_ direct space and *D*_max_ in Table 1) confirmed the slightly smaller dimensions of the NSD3-PWWP2-SET construct with respect to the NSD3-SET-PHD4 one. A good agreement between real and reciprocal *R*_*g*_ values is present for each dataset. The molecular weight values estimated in Table 1 are in fair agreement with those expected based on the primary sequence (42.5 and 44.5 kDa for NSD3-SET-PHD4 and NSD3-PWWP2-SET, respectively).

#### 3.1.1 Dataset selection

Dataset selection has been performed using the quality of the *P(r)* function determination, which was assessed by considering the quality estimation score supplied by GNOM and the shape ambiguity score supplied by the AMBIMETER program (Petoukhov and Svergun, 2015), which is related to the number of shape topologies compatible with a given *P(r)* curve (Figure 1G). Their values indicate that datasets 1_1 and 2_1 are the best ones for the NSD3-PWWP2-SET and NSD3-SET-PHD4 constructs, respectively, since their representative points in the scatter plot of Figure 1G are in the region of the lowest shape ambiguity and higher fit quality. In particular, dataset 1_1 has a very low shape ambiguity score (0.82), indicating a unique *ab initio* 3D reconstruction. In contrast, dataset 2_1 has a very high fit quality (0.84, the maximum is 1), indicating a reliable estimate of the pair distribution function. Both the selected datasets correspond to samples with lower protein concentrations. They have been obtained by re-binning the SAXS profiles on a log-scale to 800 points (1_1) or joining every third point (2_1). Further indications that corroborate this choice are the following: dataset 2_1 has a lower difference between direct and reciprocal *R*_*g*_ values, and dataset 1_1 shows the lowest difference between the estimated molecular weight (43.7 kDa) and the expected one (44.5 kDa) (Table 1). From Figure 1G, it can be noted that representative points of NSD3-SET-PHD4 have a systematically lower *P(r)* quality estimation score than those of NSD3-PWWP2-SET.

#### 3.1.2 Molecular envelope determination

**FIGURE 2:**
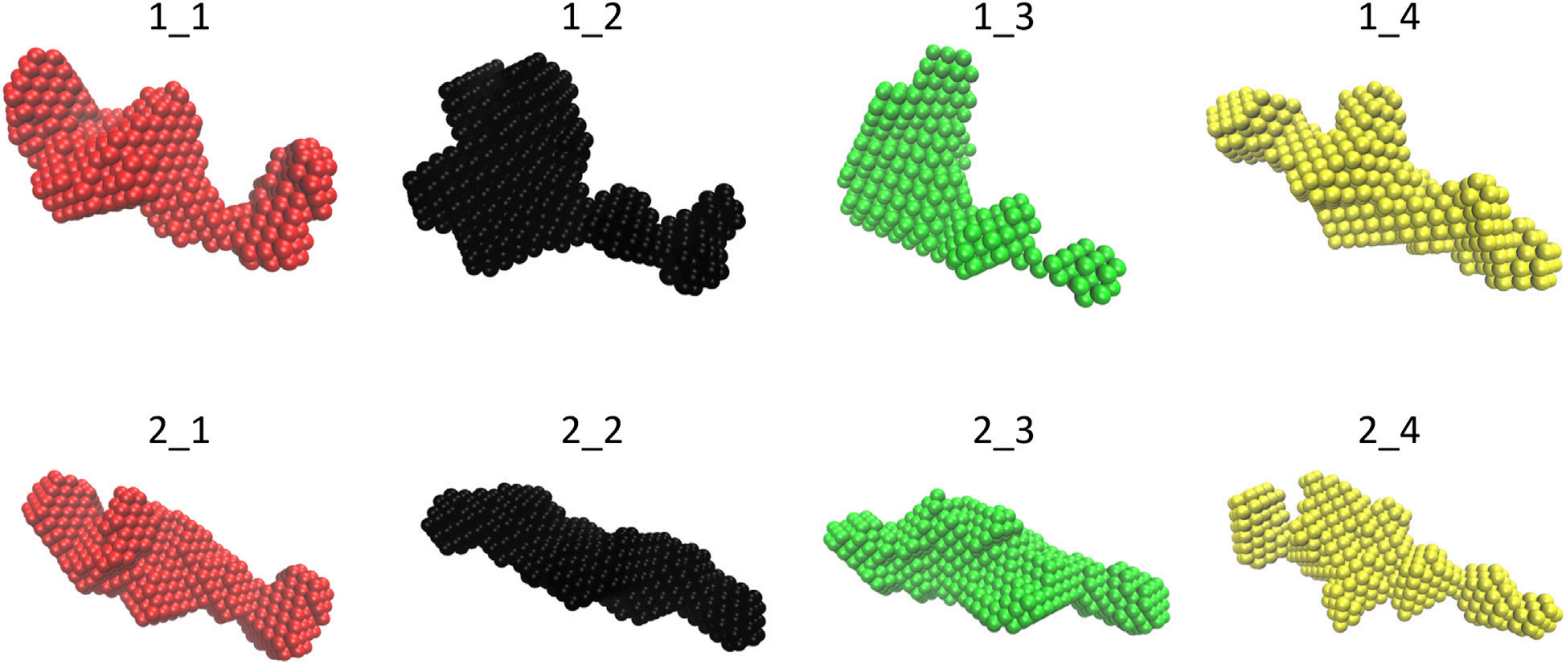
Final molecular envelope models for SEC-SAXS datasets of NSD3-PWWP2-SET (first row) and NSD3-SET-PHD4 (second row) constructs. The color code is the same as of Figure 1.

The molecular envelopes determined for each dataset are shown in Figure 2 for each dataset. They have a similar elongated shape for both constructs, apart from datasets 1_2 and 1_3, for which the superposition of the 20 envelopes calculated by DAMMIF was not optimal, which is in agreement with the fact that these datasets have the highest shape ambiguity scores (Figure 1G).

The selected SAXS data relative to the NSD3-PWWP2-SET and NSD3-SET-PHD4 samples (datasets 1_1 and 2_1, respectively) have been deposited in the SASBDB database (Kikhney et al., 2020) in entries n. SASDNL8 and SASDNK8, respectively. All individual models and fits of the molecular envelope are available in these entries as additional information.

### 3.2 Structural modeling

#### 3.2.1 Homology modeling and validation

**FIGURE 3:**
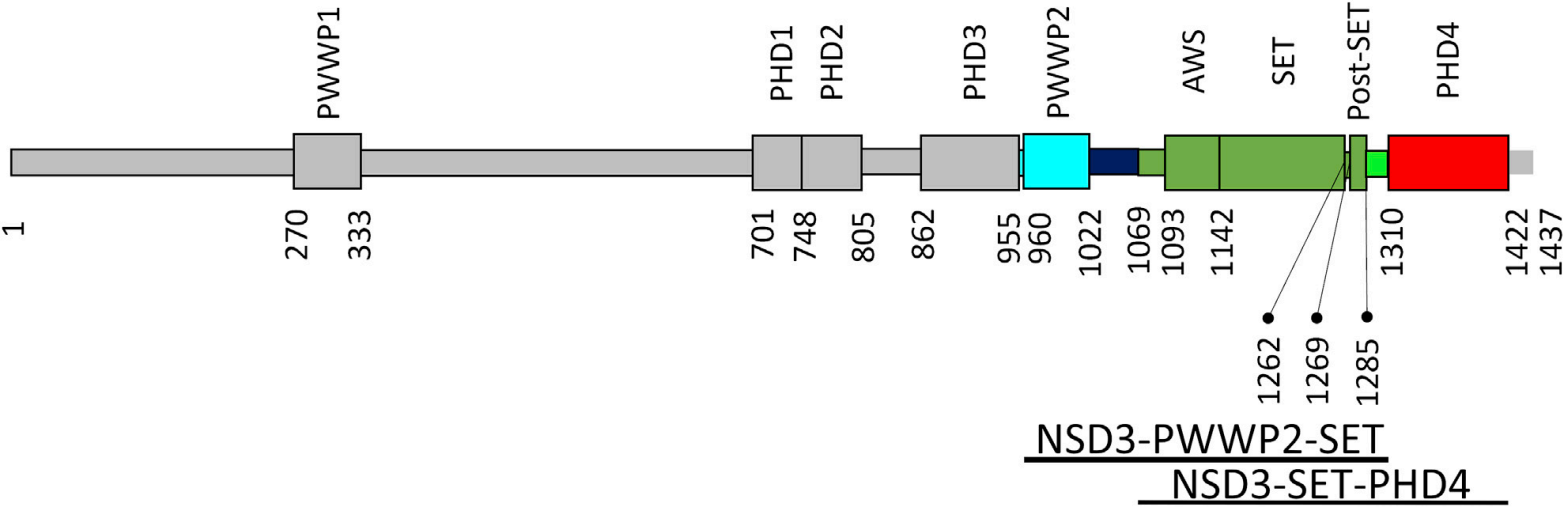
NSD3 domain organization. Domains are shown as rectangles, and those of interest for this work are represented in cyan (PWWP2), green (AWS, SET, and postSET), and red (PHD4). The numbers of residues delimiting the domains are reported together with the range of residues covered by the two constructs under investigation (bottom).

Figure 3 shows the domain organization of the whole NSD3 protein. In such a figure, the regions used in the homology modeling processes exploited in this work are colored in cyan (PWWP2), green (AWS, SET, and postSET), and red (PHD4). The models produced by the top–down modeling strategy (the one based on the entire sequence from PWWP2 to PHD4) are shown in Supplementary Figures S3A–D. Peculiar differences can be observed among the models as follows: the AlphaFold model shows the highest content of secondary structure elements (Supplementary Figure S3A), the ColabFold models constantly maintain the orientation of the PWWP2-SET and SET-PHD4 linkers concerning the SET domain (Supplementary Figure S3B), the I-Tasser models show a compact arrangement of individual domains and their linkers (Supplementary Figure S3D), and RaptorX provides a large variability in the orientation of PWWP and PHD4 domains with respect to the SET domain (Supplementary Figure S3C).

In the case of the bottom–up strategy, individual domains generated by Phyre2 (Supplementary Figure S4) have been used to build NSD3 models able to fit the envelopes of selected SAXS datasets, i.e. 1_1, related to NSD3-PWWP-SET injected at 0.6 mg/mL, and 2_1, related to NSD3-SET-PHD4 injected at 1.0 mg/L. Although such a strategy allows using SAXS data from an early stage, it has the drawback that it does not guarantee the overlap between the common region between the two NSD3 constructs, as occurred in the case of top–down modeling (Supplementary Figure S3E).

**FIGURE 4:**
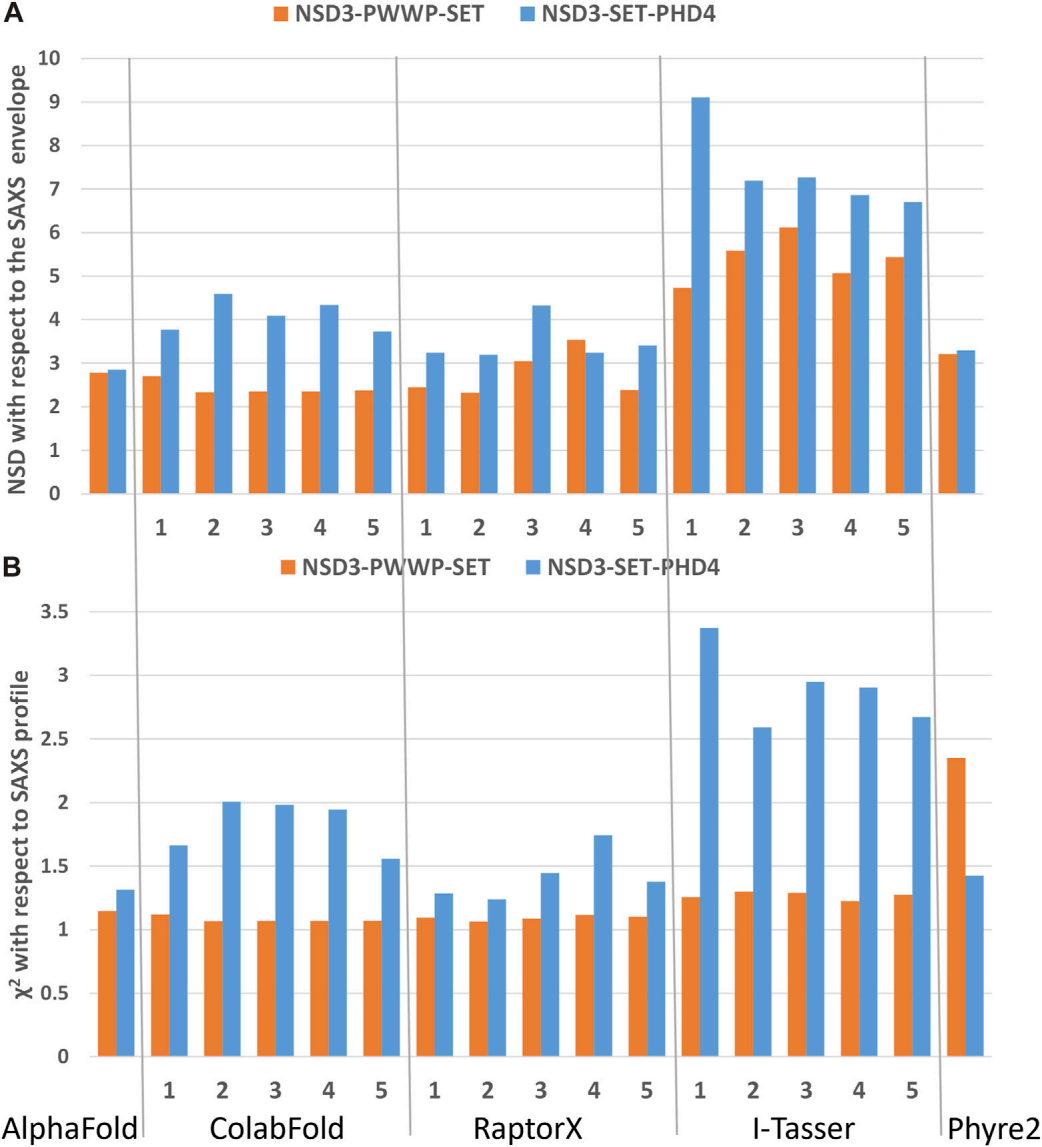
Validation of the homology models by means of SAXS data on the dataset 1_1 (NSD3-PWWP2-SET injected at 0.6 mg/mL) and 2_1 (NSD3-SET-PHD4 injected at 1.0 mg/mL) in reciprocal **(A)** and direct **(B)** spaces. Predictions of web servers AlphaFold, ColabFold, RaptorX, I-Tasser, and Phyre2 have been assessed by fitting them with SAXS data in reciprocal space **(A)** and by measuring their normalized spatial discrepancy (NSD) with respect to the corresponding SAXS molecular envelopes in direct space **(B)**. The validation parameters NSD and *χ* ^*2*^ obtained for each generated model are shown.

**FIGURE 5:**
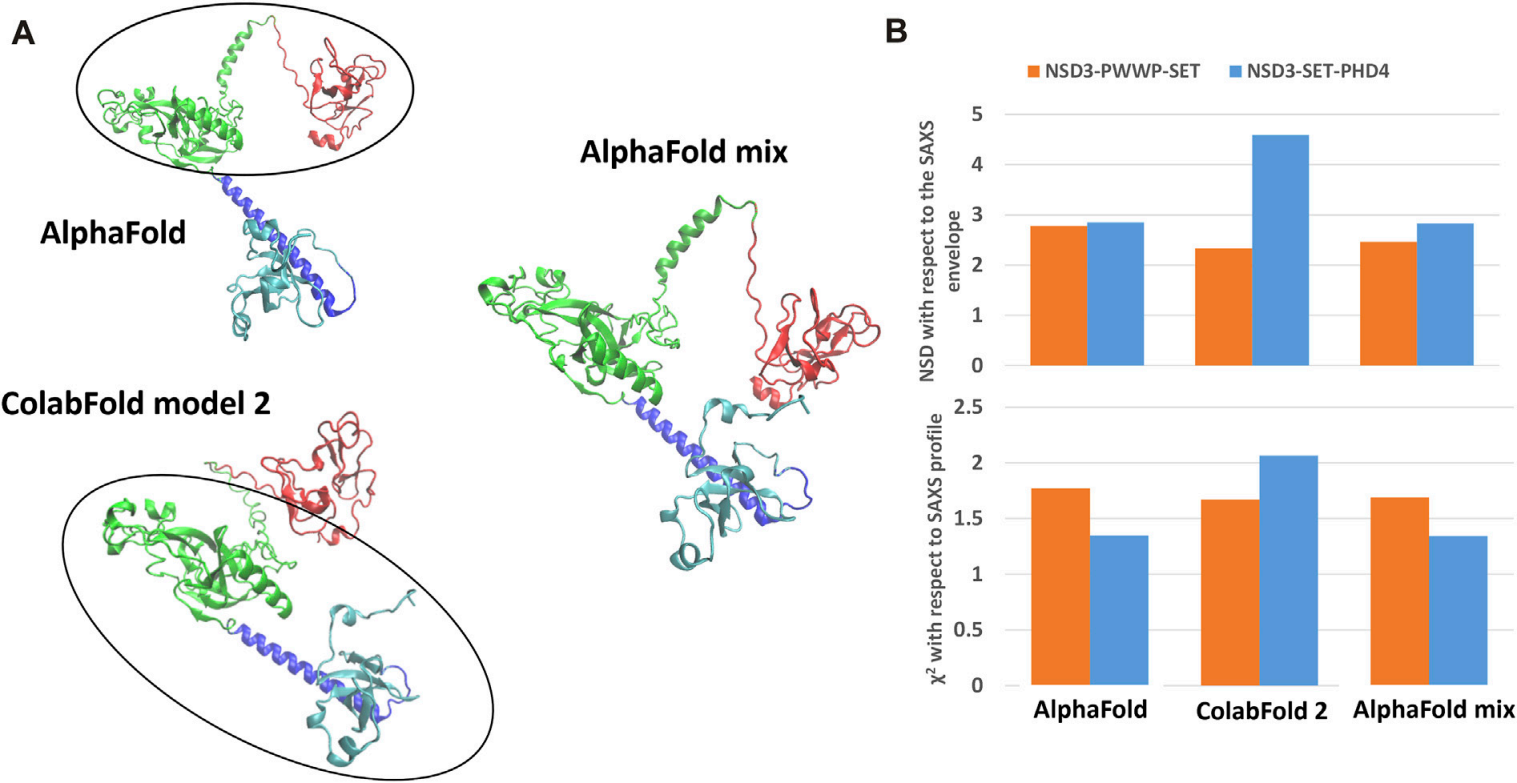
Structural model obtained by combining the NSD3-SET-PHD4 region of the AlphaFold model with the NSD3-PWWP-SET region of the ColabFold model 2 **(A)**. Values of validation parameters NSD and *χ* ^*2*^ obtained for the original models and the mixed one, separately considering the NSD3-PWWP-SET and NSD3-SET-PHD4 regions **(B)**.

The best predictions resulting from such modeling processes (those showing the lowest *χ* ^*2*^ against SAXS data and normalized spatial discrepancy values against the SAXS envelope) for both constructs have been obtained for the AlphaFold model (Figure 4). Second, there are the first two models generated by RaptorX, which mainly differ in how the linkers are structured and in the plane in which they interact (they are rotated by about 90°, as shown in Supplementary Figure S3C). The compact configuration of the I-Tasser models is a systematic disagreement with SAXS. Considering the two constructs in Figure 4 separately, it is worth noting that NSD3-SET-PHD4 has the lowest *χ* ^*2*^ and NSD scores for AlphaFold, while NSD3-PWWP2-SET is best modeled by the ColabFold model 2. Based on this evidence, we have created an AlphaFold mixed model by combining the best regions from the two models, considering the common region among the two constructs as the lever arm for the superposition (Figure 5A). Notably, this operation brings the PWWP and PHD4 domains close to each other, although they were far away in the two starting models. As expected, the validation parameters of the so-obtained mixed model are improved with respect to the original models (Figure 5B).

A further approach to generate an atomistic model of the NSD3 C-terminal involved the use of CORAL to place individual domains, as predicted by AlphaFold, guided by the agreement with the SAXS profile. This procedure is heavily influenced by the choice of even loose restraints about contacting residues. The best model obtained by combining the results of the procedure applied separately to the NSD3-PWWP2-SET and NSD3-SET-PHD4 regions is shown in Supplementary Figure S5 together with the related validation parameters.

#### 3.2.2 Optimization of the best models against SAXS data

The best homology model was refined against SAXS data by making them flexible through molecular dynamics (MD). Experimental data were included in the simulation using the technique known as molecular dynamics flexible fitting (MDFF), where the MD is restrained by the experimental molecular envelope, which represents an additional potential that drives the simulation. An additional restraint from high-resolution data from X-ray diffraction or NMR was introduced using the targeted molecular dynamics approach, which was applied to the PWWP, SET, and PHD4 domains, considering their respective experimental structures as targets. The SAXS restraints were not applied separately to NSD3-PWWP2-SET and NSD3-SET-PHD4 regions since this would have led to final models of the two regions that are not compatible with each other and would have involved performing MD on partial models, leading to approximate results. Instead, the SAXS restraints were applied by overlapping them on the initial conformation of the homology model. In this way, the two experimental envelopes of the two constructs were combined to form a unique restraint that can be used for local optimization of the whole homology model driven by MD.

The MDFF procedure was applied to the AlphaFold mixed model, which showed the best validation parameters among the full-atom models generated. For comparison, it was also applied to the AlphaFold model and the RaptorX model 1 (the latter was preferred to the RaptorX model 2, which shows a similar mutual positioning of the PWWP and PHD4 domains because it holds a more structured linker between PWWP and SET). Instead, it was not possible to apply the MDFF procedure to the CORAL model due to the incomplete modeling of their linkers.

**FIGURE 6:**
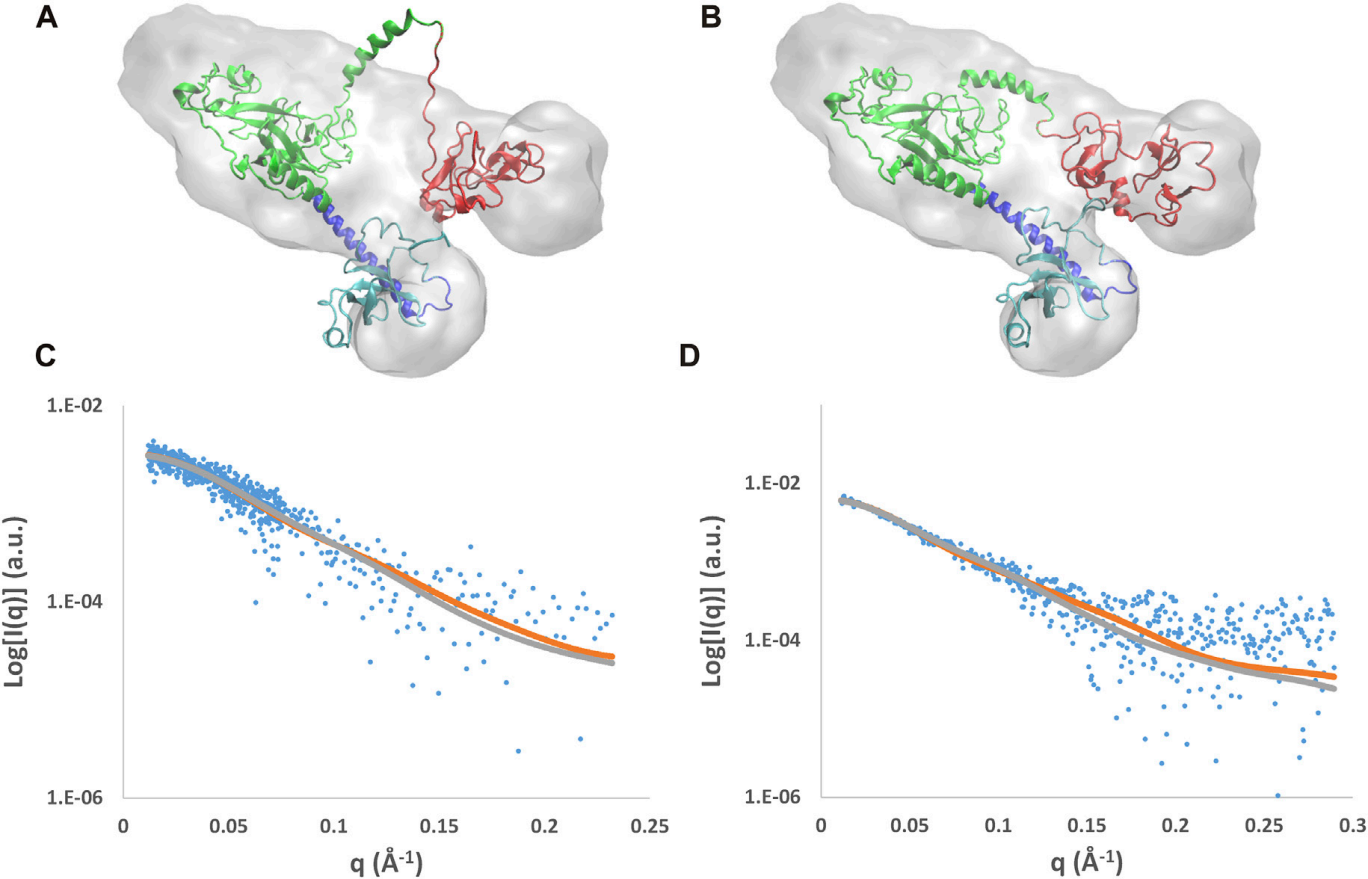
Results of the MDFF optimization applied to the AlphaFold mix model. Initial **(A)** and final **(B)** models superposed to the molecular envelope calculated from SAXS data and their fit with SAXS profiles for the NSD3-PWWP2-SET **(C)** and NSD3-SET-PHD4 **(D)** regions. The molecular envelope is shown as the transparent gray surface, and the models are shown in graphical representation, with the following color code: PWWP2 (cyan), PWWP2-SET linker (blue), SET (green), and PHD4 (red). Observed SAXS profiles (blue dots) and those calculated before (gray line) and after (brown line) application of MDFF are shown.

Results of the MDFF optimization of the AlphaFold mixed model are reported in Figure 6, where the model conformations before and after the MDFF run are shown together with the experimental molecular envelopes applied as a restraint during the simulation. The initial model partially covered by the envelope (Figure 6A) is well-fitted within it at the end of the simulation (Figure 6B), where the biggest variations concern the linker between SET and PHD4. As a result, the cross-correlation coefficient between the experimental and calculated envelopes (CORR) and the mean C_α_ deviation with respect to the initial model (RMSD) both increase during the MDFF run (Supplementary Figures S6A and B). Considering the NSD3-PWWP2-SET and NSD3-SET-PHD4 regions separately, it can be found that the first slightly decreases its size, while the second increases it by about 0.5 Å (Supplementary Figures S6C and D). The direction of these changes is consistent with the information given by the experimental assessment of the radius of gyration (Table 1), since the *R*_*g*_ of the NSD3-PWWP2-SET region of the AlphaFold model (35.0 Å) is above its SAXS-derived value in direct space (33.3 Å), while the contrary occurs for the *R*_*g*_ of the NSD3-SET-PHD4 region (32.9 Å of AlphaFold model *versus* 36.6 Å for the experimental value). In the reciprocal space, the initial and final models, considered separately for the two regions, produce different calculated SAXS profiles (Figures 6C and D). The *a posteriori* assessment of the agreement between the experimental and calculated SAXS profiles as a function of the simulation time (Supplementary Figures S6C and D) indicates that the simulation rapidly converges toward best models, reaching *χ* ^*2*^ values of 1.07 for NSD3-PWWP2-SET and 1.23 for NSD3-SET-PHD4.

Analogous results are obtained by applying the MDFF optimization to the AlphaFold model (Supplementary Figure S7), although a higher value of *χ* ^*2*^ (1.74) is reached for the NSD3-PWWP-SET region with respect to the AlphaFold mix model. Instead, in the MDFF optimization of the RaptorX model 1, a better fit of the model in the direct space does not turn into an overall improvement of the model in the reciprocal space. In particular, the NSD3-SET-PHD4 region has an opposite behavior with respect to the previous cases as it decreases its radius of gyration while increasing the *χ* ^*2*^ of the fit (Supplementary Figure S8).

#### 3.2.3 Comparative analysis of the generated models

A comparative analysis of the structural solutions obtained was performed by considering the structural diversity, as measured by the residue-by-residue backbone dihedral angles, and the agreement of the model with SAXS data, which was assessed in the direct space by the normalized structural discrepancy with the *ab initio* molecular envelope and in the reciprocal space by the *χ* ^*2*^ of the fit with the SAXS profile. This analysis, detailed in Supplementary Material (Supplementary Figures S9, S10), indicates that the structural variations introduced by MDFF are not covered by other homology modeling tools and that the solution obtained by MDFF on the AlphaFold mix model is the best one since the agreement with SAXS data is improved in both the NSD3-PWWP2-SET and NSD3-SET-PHD4 regions. The resulting model shows better agreement with SAXS data than those generated by AlphaFold, Raptor X (model 1), or even CORAL.

The AlphaFold-derived models optimized by MDFF relative to the selected NSD3-PWWP2-SET and NSD3-SET-PHD4 samples have been deposited in the SASBDB entries n. SASDNL8 and SASDNK8, respectively.

#### 3.2.4 Analysis of the full-length C-terminal model

**FIGURE 7:**
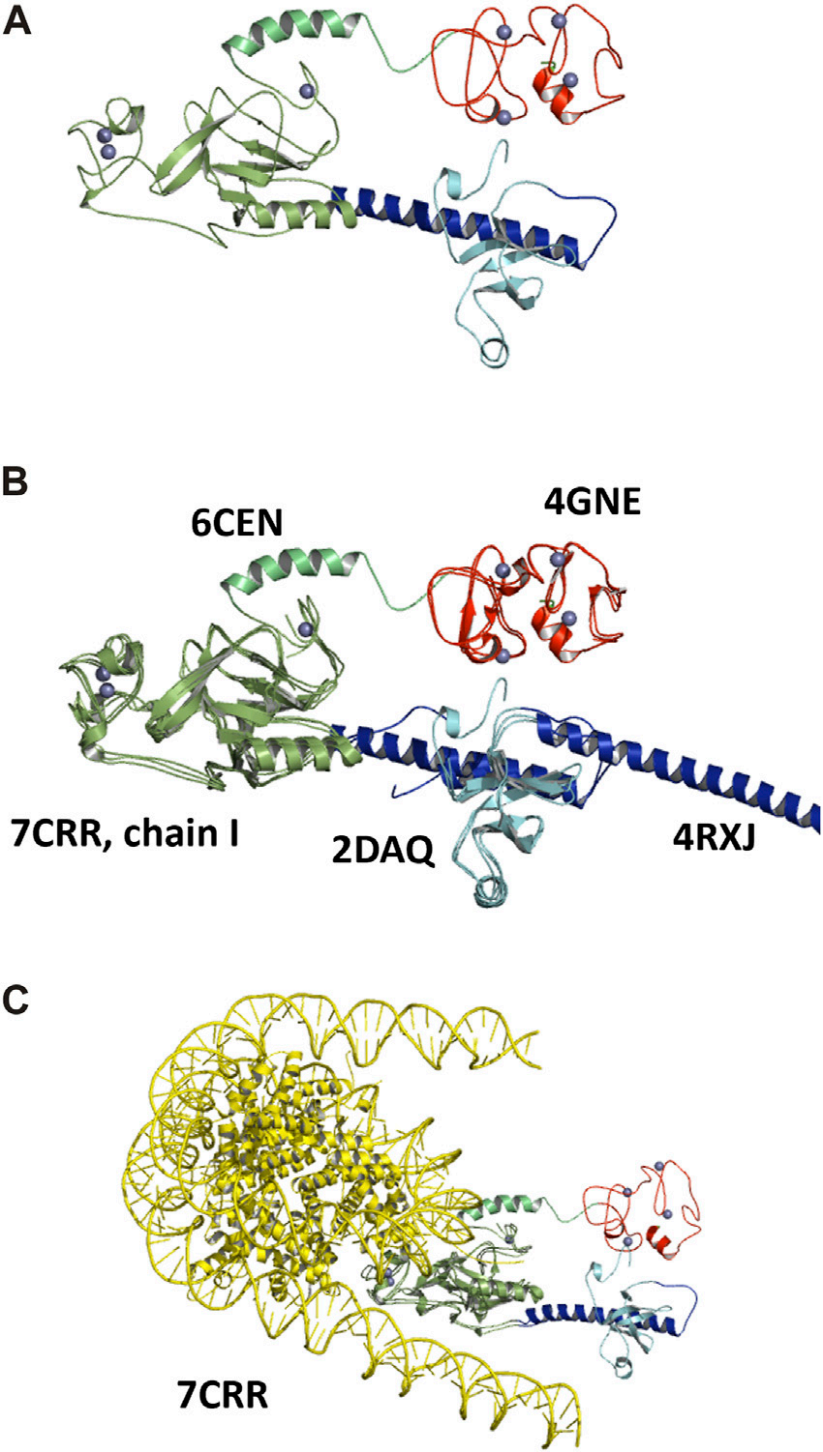
Structural models for the NSD3-PWWP2-SET and NSD3-SET-PHD4 regions obtained by the MDFF optimization of the AlphaFold mix model, shown in graphical representation with the following color code: PWWP2 (cyan), PWWP2-SET linker (blue), SET (dark green), SET-PHD4 linker (light green), and PHD4 (red). Zn ions are shown as gray spheres **(A)**. The same model is shown superposed to the crystal structures 6CEN, 4GNE, and 4RXJ to the NMR model 2DAQ and the chain I of the cryo-EM structure 7CRR **(B)**, and the entire structure 7CRR, comprising histones and nucleosomal DNA (yellow) **(C)**.

The added-value of this structural investigation is to supply a complete characterization of the NSD3 C-terminal region comprising the PWWP2, SET, and PHD4 domains. The most plausible model, i.e., the one obtained by the MDFF refinement applied to the AlphaFold mix model, is given in Figure 7A and confirms the presence of a fully structured linker between PWWP and SET and a partially structured linker between SET and PHD4, where an α-helix is present in the residues ranging from 1,292 to 1,311.

The superposition of this model with the known structural models of individual NSD3 C-terminal domains is shown in Figure 7B. The SET domain characterized in this study is in a good overlap with that from the crystal structure with the PDB code 6CEN (RMSD = 0.9 Å over 217 aligned residues) and from the cryo-EM structure 7CRR (RMSD = 1.6 Å over 240 aligned residues); the PWWP2 domain is in fair overlap with those of the NMR model 2DAQ (RMSD = 1.0 Å over 72 aligned residues) and the crystal structure 4RXJ (RMSD = 0.9 Å over 73 aligned residues), while the PHD4 domain overlaps with the crystal structure 4GNE (RMSD = 0.9 Å over 95 aligned residues). However, none of the existing experimental structures can cover the full-length PWWP2-SET-PHD4 segment, so the mutual arrangement of individual domains can only be inferred by using the SAXS-derived structural model. It is interesting to note that the α-helix connecting the PWWP2 and SET domains actually adopts two opposing directions in the 2DAQ and 4RXJ models, so our investigation resolves this controversy by indicating 2DAQ as the model that best fits the actual conformation adopted by the helix when the full C-terminal region is considered.

The superposition of our SAXS-derived model with the cryo-EM structure 7CRR, comprising the NSD3 AWS, SET, and POST-SET domains interacting with the H3, H4, H2A, and H2B histone and the nucleosomal DNA, is shown in Figure 7C. We observe that no clashes occur between the two structures, i.e., the NSD3 C-terminal reconstructed by SAXS data is fully compatible with the high-resolution structure of the NSD3 catalytic core bound to mononucleosome. In particular, we note that alternative conformations of the NSD3-PWWP2-SET and NSD3-SET-PHD4 constructs, for example, those assumed by the CORAL model (Supplementary Figure S5A), would not be compatible with the cryo-EM structure due to clashes with the histone proteins bound to NSD3s. Thus, the proximity of the PWWP2 and PHD4 domains, a peculiar feature of the SAXS-derived model, is in line with the function performed by the protein. We can envisage that the presence of mononucleosomes could induce a conformational change of the NSD3 C-terminal that leads the PWWP2 and PHD4 domains to interact with the DNA.

## 4 Discussion

Several crystal structures of individual C-terminal domains of NSD3 are present in the Protein Data Bank. However, no structural information is available about the C-terminal region from PWWP2 to PHD4, despite many efforts to crystallize such a region. Here, we performed a structural investigation at a low resolution (>20 Å) of such a region using the SAXS technique coupled with size-exclusion chromatography and complemented by advanced computational modeling.

Two constructs whose sequences overlap for 247 residues were considered: one covering the region from PWWP2 to SET and the other related to the region from SET to PHD4 (Figure 3). Datasets obtained by measuring at different concentrations were selected based on two quality parameters: the shape ambiguity of their molecular envelope and the quality of the *P(r)* fit of their SAXS profile.

Homology modeling was performed using state-of-the-art procedures that strongly rely on machine learning approaches to predict the three-dimensional structure of the full-length NSD3 C-terminal region comprising the region from PWWP2 to PHD4. SAXS data on the individual constructs were then used for model validation and refinement. This top–down strategy has proven more effective than the bottom–up approach of building separate models of the two constructs driven by SAXS data and trying to put them together to form a full-length model.

Model validation was performed in direct and reciprocal space using the following two quality metrics: the normalized spatial discrepancy between the atomic model and the molecular envelope, and the agreement between calculated and observed SAXS profiles. This dual-space approach improved the sensitivity of the SAXS data, benchmarked the predicting tools adopted, and allowed the selection of the full-atom model of the NSD3 C-terminal that was in best agreement with the SAXS data. This model, obtained as a combination of two different models generated by AlphaFold, predicts closely spaced PWWP and PHD4 domains, a feature that is shared by two other well-scored models (RaptorX 1 and 2).

Model refinement was carried out on the full-length homology models by adopting molecular dynamics (MD) to introduce flexibility based on *a priori* physicochemical knowledge in the context of a complex fitting procedure. The SAXS-derived molecular envelope and experimental structural knowledge about individual domains were then introduced as restraints in MD. This flexible fitting approach, called MDFF, improved not only the agreement with SAXS data in direct space, ensuring better coverage of the *ab initio* molecular envelope, but also the agreement in reciprocal space, as verified by *a posteriori* fit of the SAXS profile, with those calculated from the MD frames.

A comparative analysis of the MDFF results was carried out by considering (i) the minimum spatial discrepancy with the SAXS-derived molecular envelope in direct space, (ii) the agreement between observed and calculated SAXS profiles in reciprocal space, and (iii) the mutual orientation of individual residues allowed to select of the best models for the NSD3-PWWP2-SET and NSD3-SET-PHD4 constructs and build a consistent model of the NSD3 C-terminal region that sheds light into the mutual arrangement of the PWWP2, SET, and PHD4 domains. Alternative generated models predicting different mutual orientations of PWWP2 and PHD4 domains were ruled out by this analysis, thus enforcing the evidence that these models are closely spaced, thus interacting with each other in solution. Known crystallographic, NMR, and cryo-EM structures of the PWWP2, SET, and PHD4 NSD3 domains cannot be located relative to each other without using this new SAXS-derived structural knowledge. Moreover, the structural model of the NSD3 C-terminal obtained here is compatible with the binding of NSD3 to mononucleosomes.

This study discloses the mutual arrangement of the PWWP2, SET, and PHD4 domains in the NSD3 C-terminal, which is not accessible by high-resolution structural techniques due to the intrinsic flexibility of this protein region. Such results could provide implications for the mechanism of functional diversity of NSD proteins and the underexplored biological function of the PWWP2 domain.

## Data Availability

The datasets presented in this study can be found in online repositories. The names of the repository/repositories and accession number(s) can be found at: <br>https://www.sasbdb.org/data/SASDNL8/, SASDNL8.
