# Supplementary material for: Structural insights into the C-terminus of the histone-lysine N-methyltransferase NSD3 by small-angle X-ray scattering
Source: Front Mol Biosci. 2024 Mar 7;11:1191246. doi: 10.3389/fmolb.2024.1191246 (PMC10955146; doi:10.3389/fmolb.2024.1191246)
Supplement: Supplementary file 1 [file DataSheet1.pdf]

Supplementary Material

Structural insight into the C-terminus of the histone-lysine N-methyltransferase NSD3 by small-angle X-ray scattering

Table S1. Molar extinction coefficient (ε) and molecular weight (MW) of NSD3-SET-PHD4 and NSD3-PWWP-SET constructs, calculated by the Expasy ProtParam server based on their sequence.

	NSD3-PWWP-SET	NSD3-SET-PHD4
Sequence range<br><br>(UNIPROT code Q9BZ95)	942-1318	1070-1423
ε_{280nm} (M^{-1} cm^{-1})	62100	50235
MW (kDa)	44.5	42.5

Table S2. SAXS sample, data-collection, analysis and 3D modelling details for NSD3-PWWP2-SET (SASBDB id SASDNL8) and NSD3-SET-PHD4 (SASBDB id SASDNK8).

**Model deposition**			
SASBDB id		SASDNL8	SASDNK8
**Sample details**			
Organism		Human	
Source		*E. Coli* BL21(DE3)	
Scattering particle composition		NSD3 protein from PWWP2 to SET domain	NSD3 protein from SET to PHD4 domain
Protein		Uniprot code Q9BZ95 from 942 to 1318	Uniprot code Q9BZ95 from 1070 to 1423
Sample environment/configuration	Mode	SEC-SAXS	
	Solvent composition	0.5 M NaCl, 20 mM Tris-HCl (pH 8.5), 5 mM DTT	
	Sample concentration (mg/ml)	0.6*	1.0*
	Sample temperature (°C)	20	
	In-beam sample cell	1 mm quartz capillary, flow	
**SAS data collection**			
Data-acquisition/reduction software		DAWN	
Source/instrument description		B21 beamline (Diamond Synchrotron Ltd), EIGER 4M detector (Dectris)	
Measured q-range (q_{min}–q_{max}) (Å^{-1})		0.0026-0.3400	0.0027-0.3400
Method for scaling intensities		Absolute scaling (cm^{-1}) by using water as reference	
Exposure time(s), No. of exposures		3 seconds, 1 frames	
**SAS data reduction**			
Re-binning method		Data points reduced from 2600 to 800 in a logarithmic scale in q	Data points joined every third point in a linear scale in q
**SAS-derived structural parameters**			
Method(s)/software		PRIMUSqt, AUTORg and GNOM (ATSAS ver 3.0.2)	
Guinier analysis	I(0) $\pm$ σ (cm^{-1})	0.00340$\pm$0.00007**	0.0064$\pm$0.0009**
	$R_g \pm \sigma$ (Å)	31.1$\pm$1.1	33.6$\pm$0.8
	qR_g range (datapoint range)	0.37-1.30 (72-300)	0.58-1.26 (38-90)
	Linear fit assessment (AUTORG fidelity)	0.60	0.71

PDDF/*P(r)* analysis	I(0) ± σ (cm^{-1})	0.00333±0.00006**	0.00658±0.00008**
	R_g ± σ (Å)	33.3±0.9	36.6±0.8
	D_{max} (Å)	112	132
	q range ($Å^{-1}$)	0.0119 -0.2559	0.0113-0.2329
	P(r) reciprocal-space fit: χ^2, CorMap P-value	1.08, 0.93	1.22, 0.42
**Scattering particle size**			
Method(s)/software		ScÅtter (ver. IV) for Porod volume, ExpasyProtParam server for molecular mass assessment from chemical composition, PRIUMS (ATSAS ver 3.0.2) for molecular mass assessment from Bayesian inference	
Porod volume ($Å^3$)		24834	30427
Molecular Mass estimates (Da)	From chemical composition	43242	40180
	From Bayesian inference (range, % confidence)	44400-47150, 93%	41500-46150, 91%
**Modelling**			
Methods/software		DAMMIF, DAMAVER, DAMMIN (ATSAS ver 3.0.2) for shape modeling, AlphaFold Protein Structure Database for homology modeling, and MDFF tool of NAMD (ver. 2.10) for fitting the model into the SAXS-derived molecular envelope	
Shape modelling	*q*-range for fit ($Å^{-1}$)	0.0119-0.2558	0.0113-0.2329
	Symmetry/anisotropy assumptions	P1	
	No. of individual model reconstructions	20 molecular envelopes were generated by DAMMIF, superposed and averaged. The corresponding DAMSTART model has been used as input for a final DAMMIN run.	
	χ^2, CorMap P-value	1.08, 0.93	1.22, 0.42
Atomistic modelling/software	*q*-range for fit ($Å^{-1}$)	0.0119-0.2320	0.0113-0.2894
	Symmetry/anisotropy assumptions	P1	
	No. of individual model reconstructions	1	1
	χ^2, CorMap P-value	1.07, 0.93	1.23, 0.50

* Reported concentration has been measured for the sample injected in the SEC column. The actual concentration of the sample eluted from the column is unknown.

** Reported value of I(0) has not been corrected for protein concentration because UV-VIS data, useful to determine the protein concentration for the SEC fractions under SAXS measurements, were not available at the beamline.

Table S3. Standard deviation of the R_g values for frames selected under the p2 peaks ($\sigma_{<Rg>}$).

Construct	ID	Concentration (mg/ml)	Number of selected frames	$\sigma_{<Rg>}$ (Å)
NSD3-PWWP-SET	1_4	3.8	4	0.51
	1_3	1.6	4	0.74
	1_2	1.6	5	0.14
	1_1	0.6	4	0.06
NSD3-SET-PHD4	2_4	4.3	6	0.60
	2_3	1.6	4	0.73
	2_2	1.6	3	0.64
	2_1	1.0	3	0.80

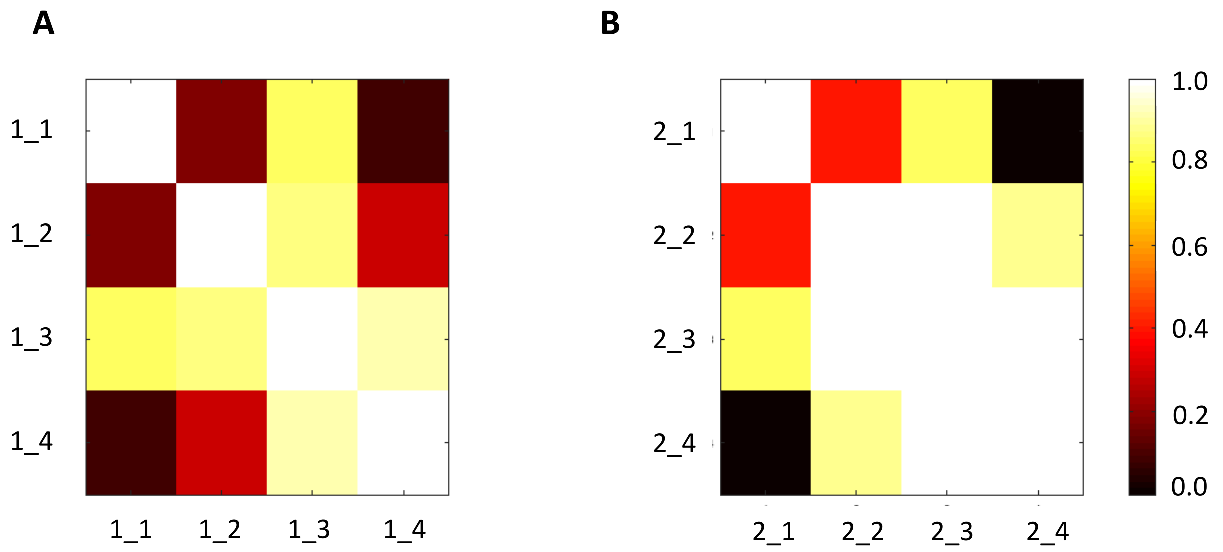

Figure S1. Similarity test among SEC-SAXS datasets by using the reduced χ^2 statistic for NSD3-PWWP2-SET (**A**) and NSD3-SET-PHD4 (**B**). The right-side bar shows colors related to p-value of the test.

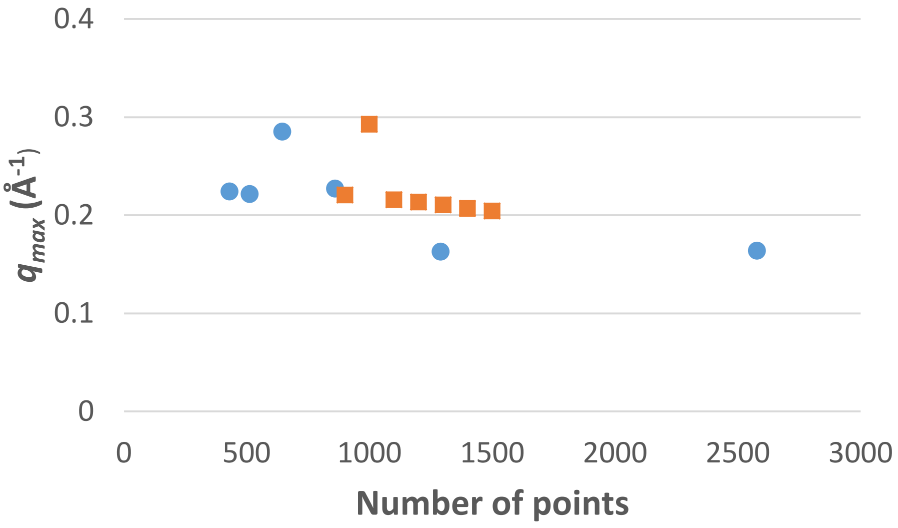

Figure S2. Results of the re-binning procedure applied to dataset 2_3. Values of q_{max} as a function of the number of points of the SAXS profile. The number of points has been reduced in linear (circles) or logarithmic (squares) way along the q axis.

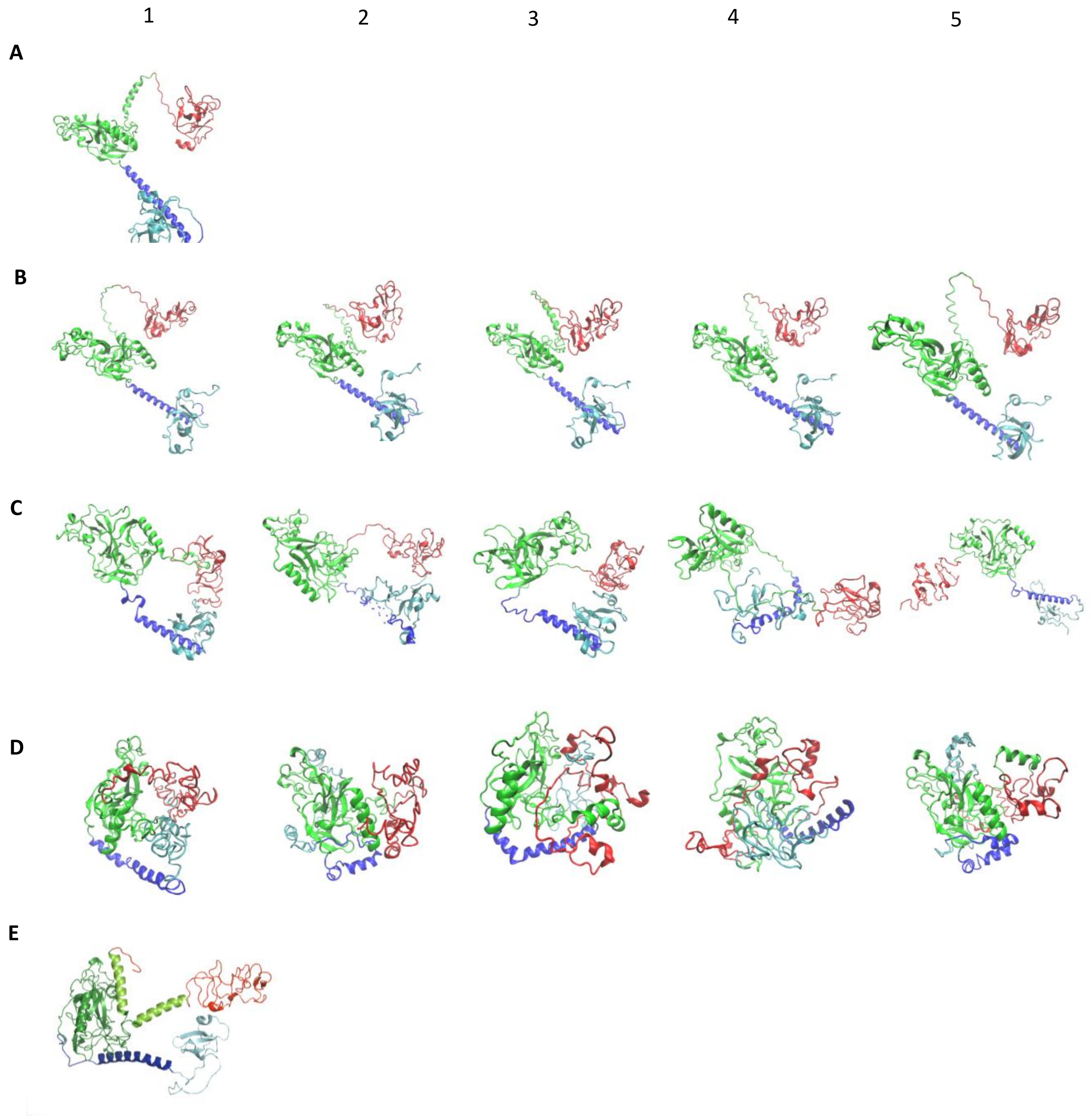

Figure S3. Structural models for the full-length NSD3 C-terminal region generated by AlphaFold (**A**), ColabFold (**B**), RaptorX (**C**), I-Tasser (**D**) and obtained starting from a Phyre2 model (**E**). Individual domains are coloured as follows: PWWP2 (cyan), PWWP2-SET linker (blue), SET (green), PHD4 (red). Numbers on the top indicate the ranking of the models supplied by the servers.

A

PWWP2-SET

GCWNCNDCKAGKKLHYKQIVWVKLGNYRWWPAEICNPRSVPLNIQGLKHDLGHDYYWVHQGRVFPYVEGDKSFAEGQTSINKTFKKA

SET-PHD4

PWWP2-SET

LEEAAKRFQELKAQRESKEALEIEKNSRKPPPYKHIKANKVIGKVQIQVADLSEIPRCNCKPADENPCGLESECLNRMLQYECHPQVCPAGD

SET-PHD4

PWWP2-SET

RCQNQCFTKRLYPDAEIIKTERRGWGLRTKRSIKKGEFVNEYVGELIDEEECRLRIKRAHENSVTNFYMLTVTKDRIIDAGPKGNYSRFMNHS

SET-PHD4

PWWP2-SET

CNPNCETQKWTVNGDVRVGLFALCDIPAGMELTFNYNLDCLGNGRTECHCGADNCSGFLGVRPKSACASTNEEKAKNAKLKQKRRKIKT

SET-PHD4

PWWP2-SET

EPKQMHEDYCFQCGDGGELVMCDKKDCPKAYHLLCLNLTQPPYGKWECPWHQCDECSSAAVSFCEFCPHSFCKDHEKGALVPSALEGRL

SET-PHD4

CCSEHDPMAPVSPEYWSKIK

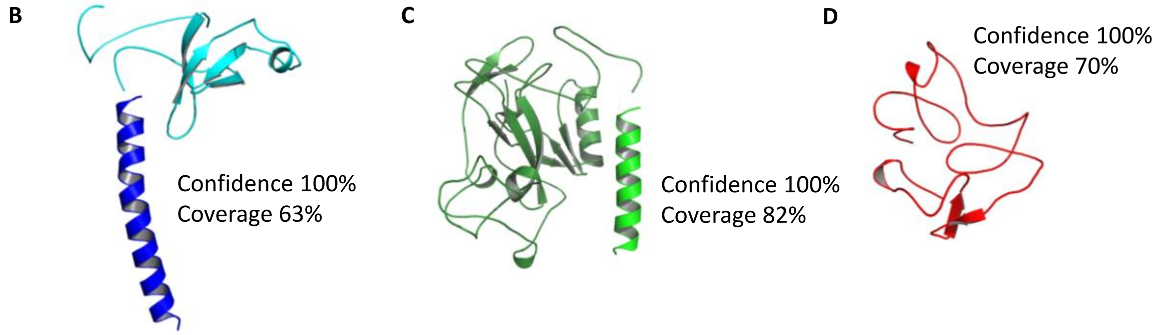

Figure S4. Models of individual domains of the C-terminal region of NSD3 obtained by homology modeling (Phyre2 server). Sequences for NSD3-PWWP2-SET (solid line) and NSD3-SET-PHD4 (shaded line) constructs (**A**). Homology models: (**B**) PWWP core (cyan) and helix connecting PWWP and SET (blue); (**C**) SET core (dark green) and helix connecting SET and PHD4 (light green); (**D**) PHD4 (red). The confidence and coverage of the homology models are shown. Each model has been produced with 100% of confidence by using the 2DAQ, 3OOI, and 4GND crystal structures as templates (respectively the NMR solution of the NSD1 PWWP2 domain, the crystal structure of the NSD1 SET domain and the crystal structure of the NSD3 PHD4-CSHCH domain). The homology models cover 63% of the input sequence in the case of PWWP2 domain and the link connecting PWWP2 and SET domains (**B**), 82% in the case of SET core and the link connecting SET and PHD4 domains (**C**), and 70% in the case of PHD4 domain (**D**). Phyre2 suggests an α-helix folding of the two linker regions PWWP2-SET and SET PHD4 (**B** and **C**, in blue and light green color respectively).

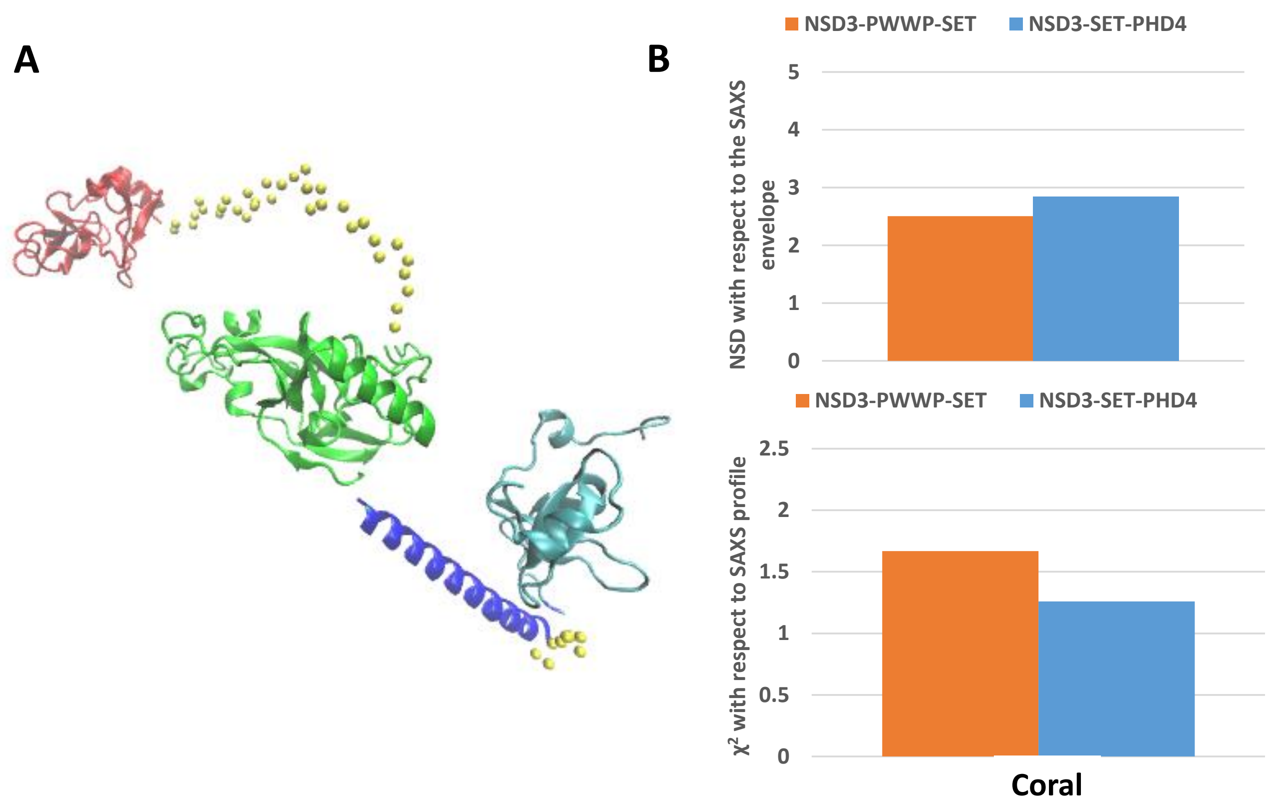

Figure S5. Structural model obtained by superimposing the results obtained for the NSD3-PWWP-SET and NSD3-SET-PHD4 datasets by CORAL, considering individual domains PWWP2 (cyan), PWWP2-SET linker (blue), SET (green), PHD4 (red) as rigid bodies, together with linkers constituted by C_α atoms, colored in yellow (**A**). Values of validation parameters NSD and χ^2 obtained for the CORAL model, considering separately the NSD3-PWWP-SET and NSD3-SET-PHD4 regions (**B**).

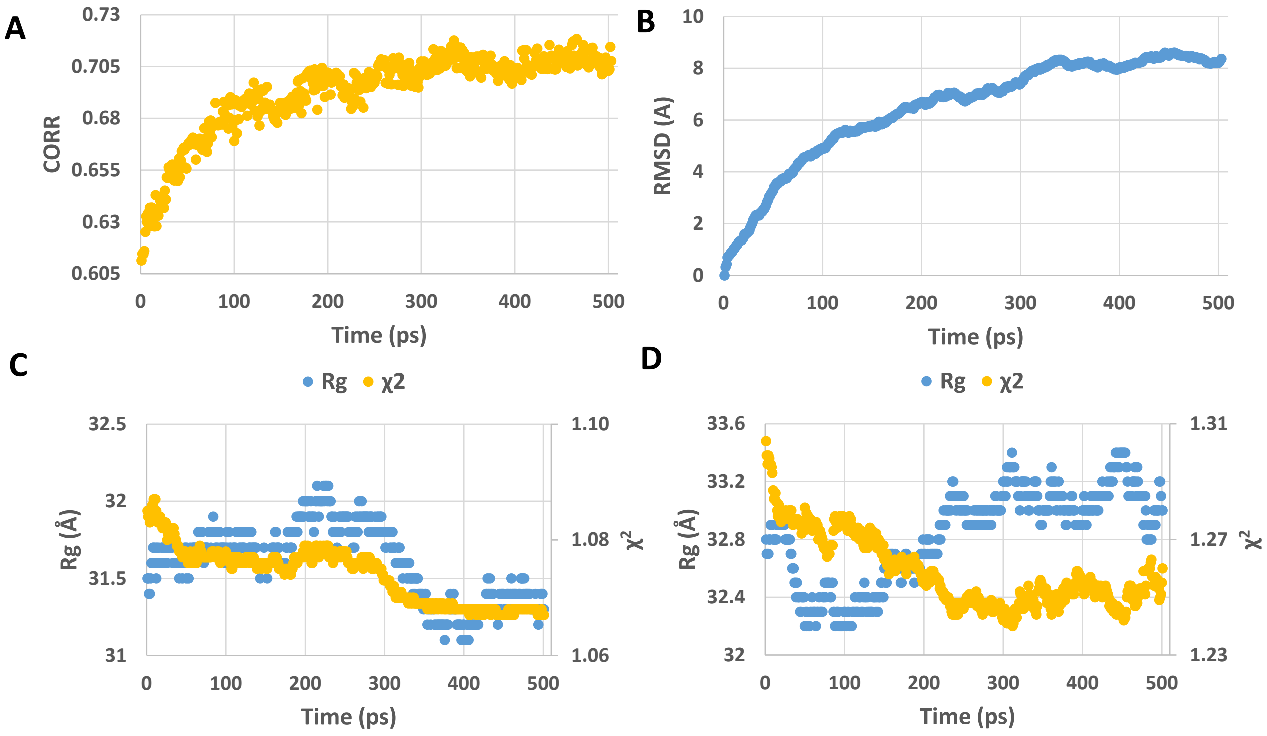

Figure S6. Results of the MDFF optimization applied to the AlphaFold mix model. The cross-correlation coefficient (CORR) between model and the experimental molecular envelope (**A**), the root mean square deviation (RMSD) of C_α atoms with respect to the initial structure (**B**), the radius of gyration of the model (R_g) and the χ^2 between experimental and calculated SAXS profiles for NSD3-PWWP2-SET (**C**) and NSD3-SET-PHD4 (**D**) constructs are reported as a function of the simulation time. The structural model obtained after 453 ps was chosen as that in best agreement with SAXS data.

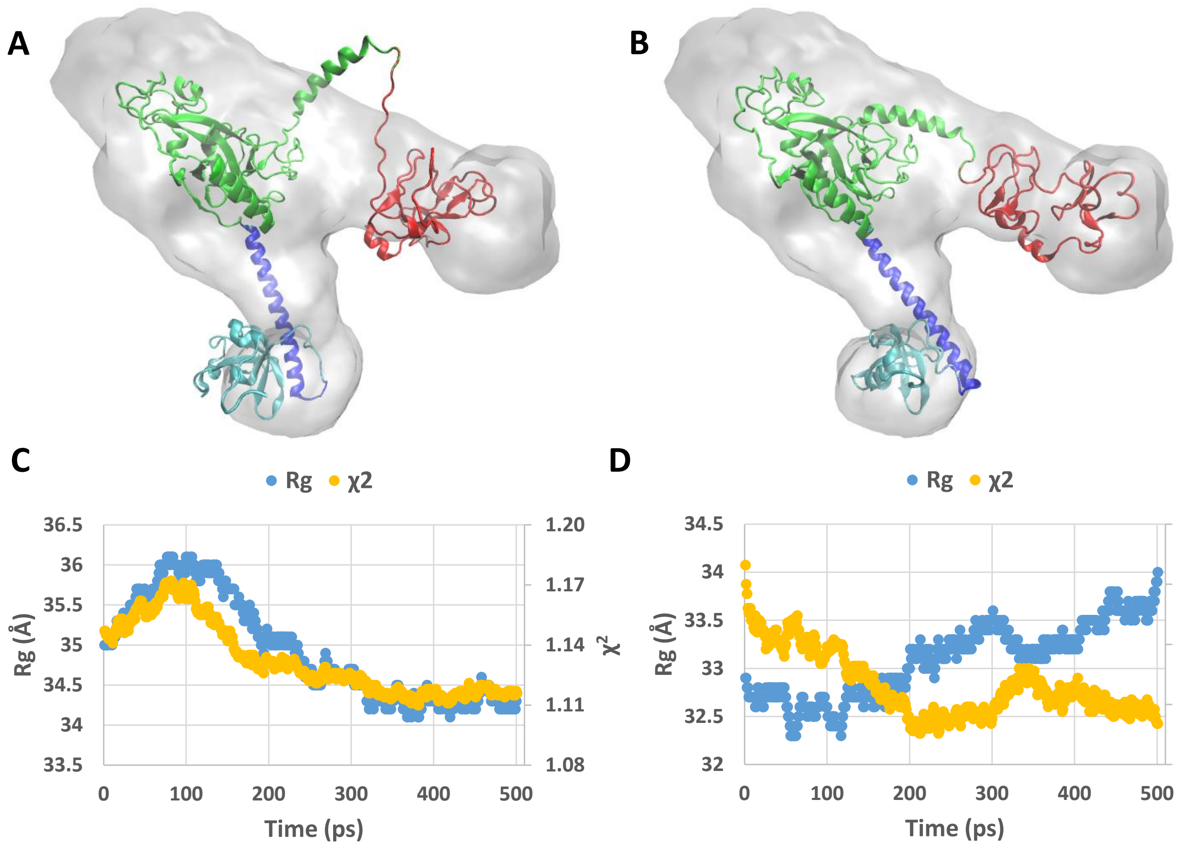

Figure S7. Results of the MDFF optimization applied to the AlphaFold model. Initial (**A**) and final (**B**) models superposed to the molecular envelope calculated from SAXS data. The molecular envelope is shown as transparent gray surface and the models are shown in cartoon representation, with the following color code: PWWP2 (cyan), PWWP2-SET linker (blue), SET (green), PHD4 (red). The radius of gyration of the model (R_g) and the χ^2 between experimental and calculated SAXS profiles for NSD3-PWWP2-SET (**C**) and NSD3-SET-PHD4 (**D**) constructs are reported as a function of the simulation time.

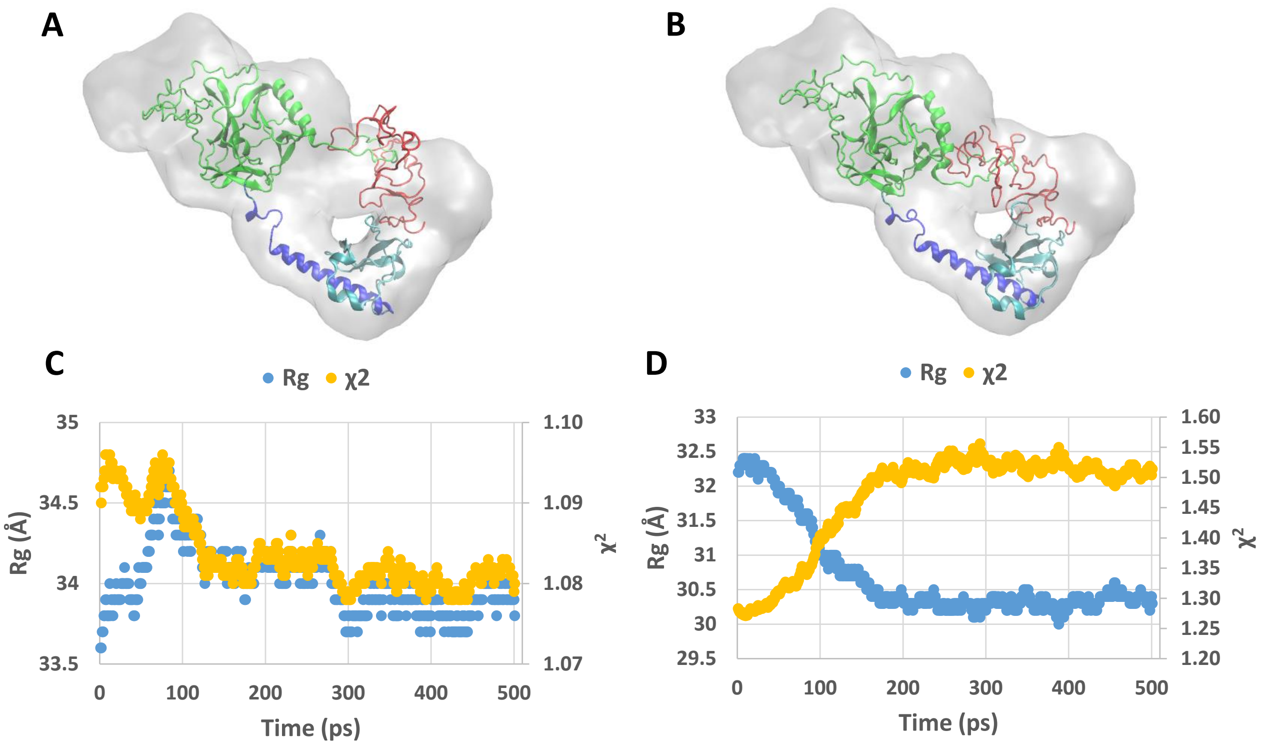

Figure S8. Results of the MDFF optimization applied to the RaptorX model 1. Initial (**A**) and final (**B**) models superposed to the molecular envelope calculated from SAXS data. The molecular envelope is shown as transparent gray surface and the models are shown in cartoon representation, with the following color code: PWWP2 (cyan), PWWP2-SET linker (blue), SET (green), PHD4 (red).The radius of gyration of the model (R_g) and the χ^2 between experimental and calculated SAXS profiles for NSD3-PWWP2-SET (**C**) and NSD3-SET-PHD4 (**D**) constructs are reported as a function of the simulation time.

Comparative analysis of the structural models

A structural comparison of the homology models generated and optimized against SAXS data has been carried out by considering unidimensional profiles of Protein Angular Value (PAV), a geometrical descriptor representing the orientation of the individual profiles along the backbone. They are shown shifted one respect to the other and colored according to the method used for their generation in Figure S9 **A**,**C**. It can be noted that the α-helix of the SET domain can be easily recognized in the PAV profiles of all structural models. Indeed, this region, which ranges between 1181 and 1195, shows very small fluctuations of PAV around an average value of 100°, which is typical for backbone dihedral angles of residues forming a α-helix. Just as easily, the linker connecting PWWP and SET domains (1026-1056) (blue) can be recognized as an α-helix in most of the models, while the linker between SET and PHD4 domains (1290-1310) forms an α-helix only in the AlphaFold, ColabFold and Phyre2 models, while it forms a loop in the I-Tasser and RaptorX models. Application of Principal Component Analysis (PCA) allows to trace the structural differences among models as distances among points representative of the PAV profiles in the score plot of the first two principal components (Figure S9 **C**,**D)**. In Figure S9 **A**,**B** all the models are considered for comparison, showing that those generated by I-Tasser and RaptorX differ substantially from the others, especially in the linker between SET and PHD4. Their representative points are clearly separated in Figure S9 **B** along the first principal component (PC1) for I-Tasser and along the second principal component (PC2) for RaptorX. In Figure S9 **C**,**D** the I-Tasser and RaptorX models are excluded from the comparison, in order to focus on the fine differences among remaining models. The ColabFold models form a well-defined cluster in Figure S9 **D**, together with the AlphaFold model and the related AlphaFold mix model. The Phyre2 model is separated along PC1, while the AlphaFold and AlphaFold mix models optimized by MDFF are separated along PC2. This suggests that the structural differences introduced by the MDFF refinement are not covered by using homology modeling procedures, so that the resulting model is intrinsically different from all those generated in this study.

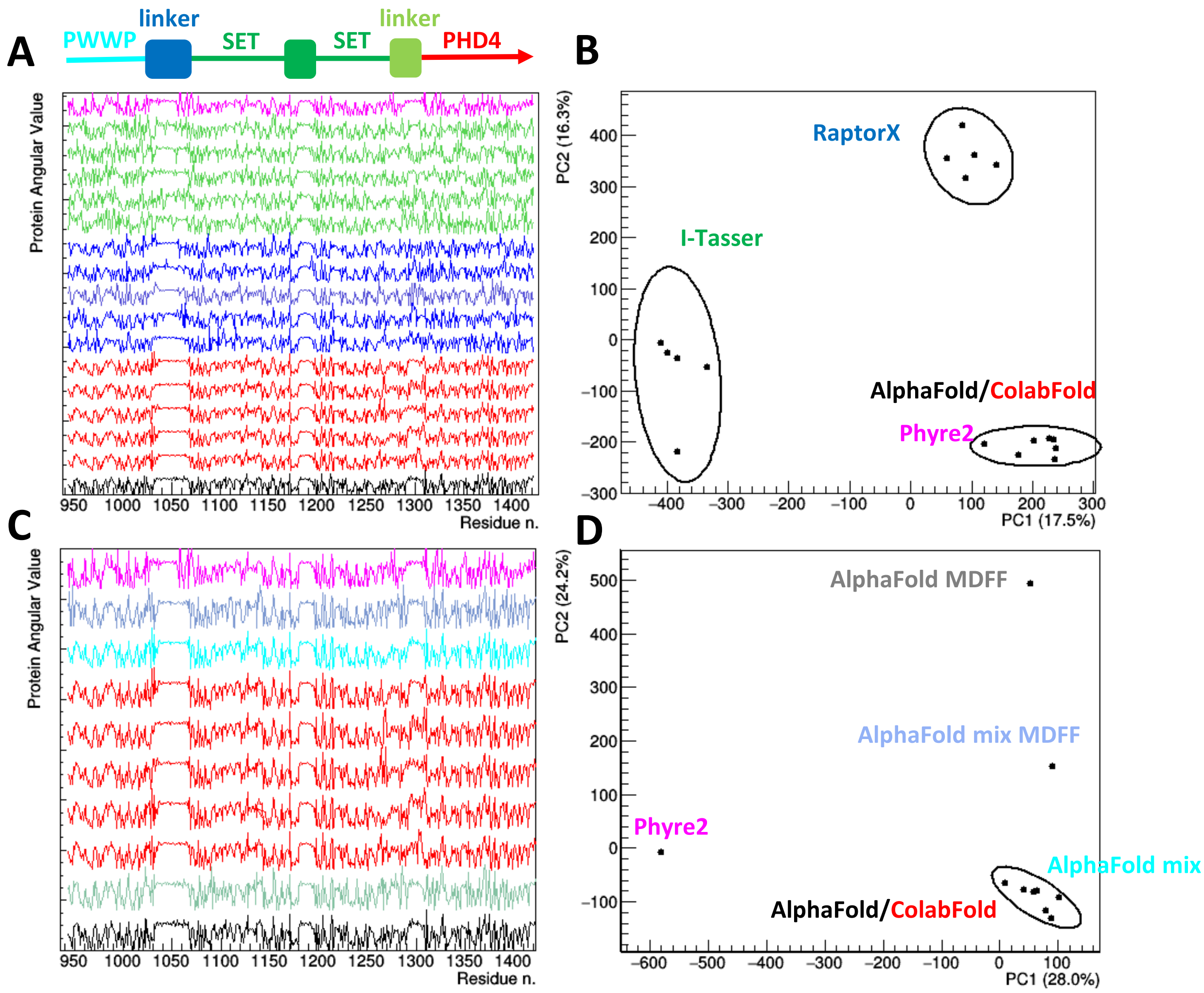

Figure S9. Comparative analysis of the Protein Angular Value (PAV) profiles, representing the conformations of the structural models generated in this study. (**A**,**C**) PAV profiles calculated from models generated in this study. A sketch of the domains of the constructs is given on the top, where predicted α-helices are shown by full boxes. (**B**,**D**) Score plot obtained by principal component analysis applied to PAV profiles, where each point represents a PAV profile. The data variance explained by the first (PC1) and second (PC2) principal components is shown on the axes. 85% confidence level ellipses indicate the results of a hierarchical clustering procedure applied to representative points in the score plot. In **C** and **D** the RaptorX and I-Tasser models have been excluded from the comparative analysis.

The effect of the application of MDFF on the full-length models in best agreement with SAXS data i.e. AlphaFold, AlphaFold mix and RaptorX model 1 (RaptorX 1) has been assessed both in the direct space, measured by the normalized structural discrepancy between model and *ab initio* molecular envelope, and in the reciprocal space, measured by the χ^2 of the fit of scattering experimental data with that calculated by the model. Results, reported in Figure S10, show as a general trend that MDFF optimization improves the agreement of the models with SAXS data both in direct and reciprocal space. However, while for AlphaFold and AlphaFold mix the improvement occurs in the same way for NSD3-PWWP-SET and NSD3-SET-PHD4 regions, for RaptorX 1 it only occurs for NSD3-PWWP-SET, while the NSD3-SET-PHD4 region worsens greatly. As a result, the model with best agreement with SAXS data is the AlphaFold mix refined by MDFF.

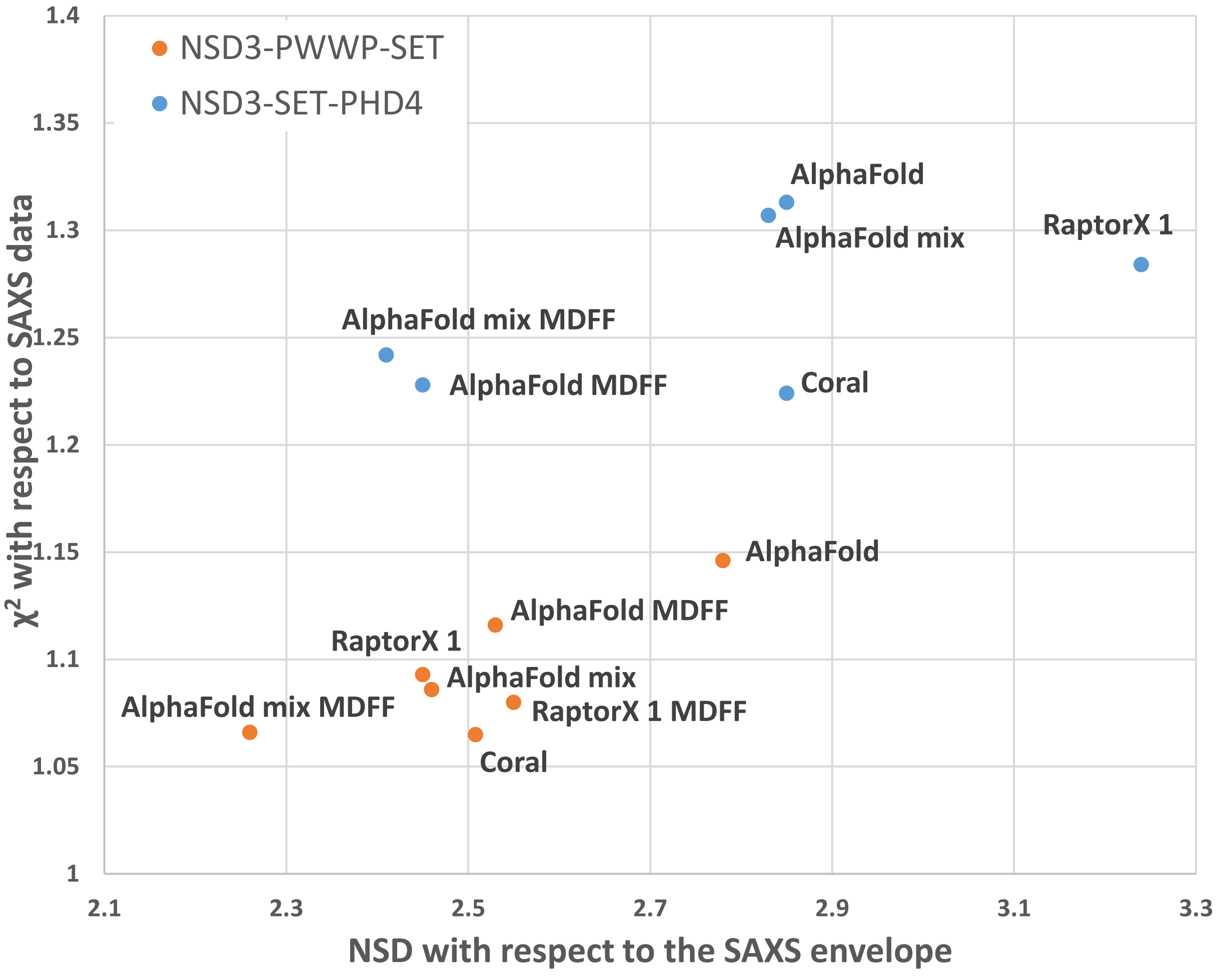

Figure S10. Assessment of the agreement of the best structural models generated by AlphaFold (AlphaFold and AlphaFold mix), RaptorX (model 1) and CORAL against SAXS data obtained by using NSD3-PWWP2-SET (blue points) and NSD3-SET-PHD4 (orange points) samples. Quality parameters are the normalized structural discrepancy (NSD) with respect to the ab initio SAXS molecular envelope (horizontal axis) and the χ^2 of the fit with SAXS profiles (vertical axis). Point labels refer to the original homology models and those optimized by MDFF.
